# α-Synuclein fibril-specific nanobody reduces prion-like α-synuclein spreading in mice

**DOI:** 10.1038/s41467-022-31787-2

**Published:** 2022-07-19

**Authors:** Yemima R. Butler, Yuqing Liu, Ramhari Kumbhar, Peiran Zhao, Kundlik Gadhave, Ning Wang, Yanmei Li, Xiaobo Mao, Wenjing Wang

**Affiliations:** 1 Life Sciences Institute, University of Michigan, Ann Arbor, MI USA; 2 Department of Chemistry, University of Michigan, Ann Arbor, MI USA; 3 Institute for Cell Engineering, Johns Hopkins University School of Medicine, Baltimore, MD 21205 USA; 4 Department of Neurology, Johns Hopkins University School of Medicine, Baltimore, MD 21205 USA; 5 Present Address: Key Laboratory of Bioorganic Phosphorus Chemistry & Chemical Biology (Ministry of Education), Department of Chemistry, Tsinghua University, 100084 Beijing, China

**Keywords:** Protein design, Neurodegeneration

## Abstract

Pathogenic α-synuclein (α-syn) is a prion-like protein that drives the pathogenesis of Lewy Body Dementia (LBD) and Parkinson’s Disease (PD). To target pathogenic α-syn preformed fibrils (PFF), here we designed extracellular disulfide bond-free synthetic nanobody libraries in yeast. Following selection, we identified a nanobody, PFFNB2, that can specifically recognize α-syn PFF over α-syn monomers. PFFNB2 cannot inhibit the aggregation of α-syn monomer, but can significantly dissociate α-syn fibrils. Furthermore, adeno-associated virus (AAV)-encoding EGFP fused to PFFNB2 (AAV-EGFP-PFFNB2) can inhibit PFF-induced α-syn serine 129 phosphorylation (pS129) in mouse primary cortical neurons, and prevent α-syn pathology spreading to the cortex in the transgenic mice expressing human wild type (WT) α-syn by intrastriatal-PFF injection. The pS129 immunoreactivity is negatively correlated with the expression of AAV-EGFP-PFFNB2. In conclusion, PFFNB2 holds a promise for mechanistic exploration and therapeutic development in α-syn-related pathogenesis.

## Abstract (web summary)

Butler et al. selected disulfide bond-free nanobodies to target α-synuclein fibrils. Nanobody PFFNB2 was shown to disaggregate α-synuclein fibrils in vitro and inhibit α-synuclein pathology development in neuron cultures and mouse models.

## Introduction

Lewy body dementia (LBD) is one of the most common dementias, including Dementia with Lewy Body (DLB) and Parkinson’s Disease (PD) with Dementia (PDD)^1–3^. Moreover, Alzheimer’s Disease (AD) patients with Lewy body pathology exhibit a more rapid and severe cognitive decline than AD alone^4^. There is an urgent need for effective therapies for LBD. LBD is characterized by the accumulation of aggregated α-synuclein (α-syn) in the cortex. Substantial post-mortem studies by Braak et al.^5,6^ and others^7–9^ showed that misfolded α-syn is a prion-like protein, and its pathology spreads stereotypically. A single administration of recombinant α-syn preformed fibrils (PFF) can induce endogenous α-syn monomers to aggregate and subsequent cell-to-cell transmission^10–15^. Both clinical and experimental observations support the prion-like hypothesis of aggregated α-syn fibrils^16^. Thus, targeting α-syn fibrils could provide an alternative approach to study the pathogenesis and to treat LBD and related α-synucleinopathies.

Of note, α-syn pathology and propagation are observed mainly inside neurons and α-syn is more abundant in neurons than in glia cells, indicating that the intracellular α-syn significantly drives the pathogenesis^17–19^. There have been tremendous efforts on the development of antibodies against α-syn (reviewed in Vaikath et al.^20^); however, due to the large size and structural complexity, antibodies have limited penetrability through the plasma membrane^21,22^. Consequently, α-syn antibodies typically do not enter cells but only target the extracellular α-syn fibrils^23–27^, not accessible to the essential target, the intracellular pathologic α-syn species. Furthermore, antibodies are usually not functional in the reducing environment of the cytosol due to the reduction of the disulfide bonds, which are critical for the correct folding of antibodies^28^. In addition, antibody treatment could be costly and inconvenient in long-term disease progression^29^. All these gaps indicate an urgent need to generate a reagent targeting the intracellular pathogenic α-syn.

Nanobodies are single-domain antibodies with several advantages: (1) high stability^30,31^, (2) small size (15 kDa)^31,32^ and improved brain permeability^33^, and (3) suitability for intracellular expression^34,35^. Recent advances in adeno-associated virus (AAV)-based gene delivery have provided an attractive approach to continuously express recombinant proteins binding to pathogenic targets in long-term disease treatment^22,35,36^. Some nanobodies^37,38^, including NbSyn2 and NbSyn87, were designed to bind to total α-syn (both monomers and aggregates). In particular, NbSyn87 targeted to the proteasome was found to reduce α-syn pathology both in vitro and in vivo^39,40^. However, the total α-syn-targeting nanobodies may perturb the physiological function of α-syn monomers, and show reduced efficacy against α-syn pathology due to the competitive binding with α-syn monomers. Therefore, it is necessary to develop nanobodies that specifically bind to α-syn aggregates.

Nanobodies are normally screened under extracellular oxidizing conditions^41,42^ and then applied for intracellular application under reducing conditions (e.g., intracellular cytosol). In the traditional nanobody scaffold, there are two conserved cysteine residues that form a disulfide bond in oxidizing conditions (e.g., endoplasmic reticulum, Golgi, bacterial periplasm, extracellular environment, etc.), which is critical for the stabilization of a nanobody’s structural folding^31^. However, the disulfide bond can be disrupted in reducing conditions, resulting in changes in the stability, folding, and function of the nanobody in some circumstances^43^. Thus, it is desired to develop an extracellular disulfide bond-free nanobody selection platform to ensure consistent nanobody folding.

In this study, we designed extracellular disulfide bond-free synthetic nanobody libraries by mutagenesis of the two conserved cysteine residues. We generated nanobodies that preferentially bind to α-syn fibrils, but not α-syn monomers. We determined the specificity and efficacy of one nanobody in vitro and in vivo. The α-syn fibril-specific nanobody will provide a tool for pathogenesis exploration and hold promise for therapeutic application.

## Results

### Preparation of human α-syn monomers and PFF

To obtain nanobodies that preferentially bind to α-syn fibrils but not α-syn monomers, we prepared both human recombinant α-syn monomers and PFF following the established protocol^44^. Seven days after agitation, mature α-syn fibrils were generated from α-syn monomers, and then sonicated for α-syn PFF. Transmission electron microscopy (TEM) showed the short fibrillar morphology (average length 53.8 nm) of α-syn PFF, and irregular morphology of α-syn monomers (Supplementary Fig. 1a, b). α-Syn PFF and monomers were validated with a thioflavin T (ThT) fluorescence assay (Supplementary Fig. 1c)^45,46^.

### Design of the disulfide bond-free nanobody libraries by mutagenesis of the conserved cysteine residues and randomization of the binding loops

**Fig. 1:**
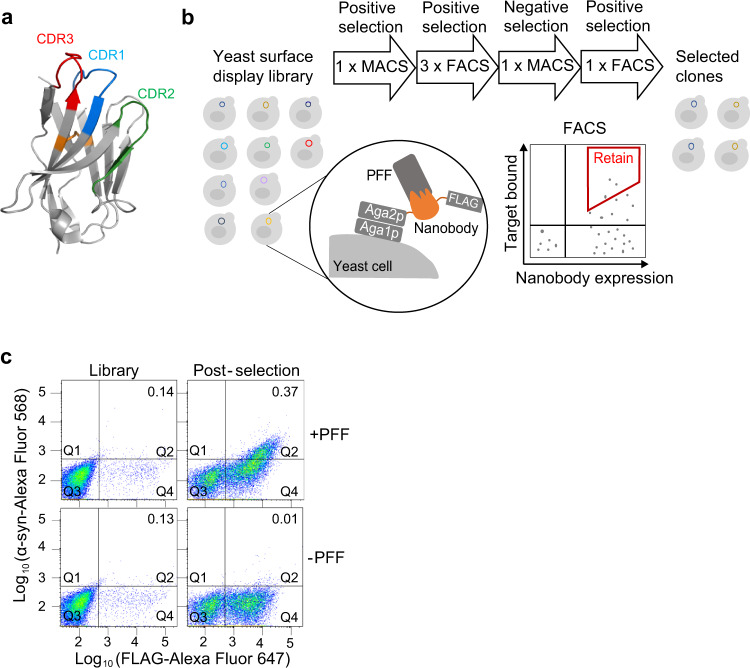
Nanobody selection against α-syn PFF. **a** Nanobody crystal structure (based on PDB: 4LDE). Conserved disulfide bond (orange) was removed in our libraries. **b** Nanobody selection schematics. Nanobodies were expressed on the yeast surface by fusion to the C-terminus of Aga2p protein, followed by the FLAG tag. Yeast cells that bind to α-syn PFF were selected using MACS and FACS. Red trapezoid, a representative selection gate. **c** FACS analysis of nanobody libraries before and after selection. After 6 rounds of selection, the selected nanobodies showed a higher PFF-binding signal, compared to the original libraries and negative control (without α-syn PFF). The numbers in the upper right Q2 indicate the yeast population ratio of Q2/Q4.

Nanobodies have a framework region consisting of β-sheets (Fig. 1a), and three variable loops that correspond to the complementary determining regions (CDR1, 2, and 3) constituting the antigen-binding site (Fig. 1a). Under oxidizing conditions, two conserved cysteine residues inside the β-sheets form a disulfide bond (orange line, Fig. 1a), stabilizing the nanobody scaffold^31^. Because the folding and function of traditional nanobodies with the disulfide bond may change in reducing conditions from oxidizing conditions, we generated nanobody libraries with the disulfide bond removed, by mutagenesis of the two conserved cysteine residues, to select nanobodies for intracellular applications.

To evaluate whether nanobodies can fold correctly and function without the conserved disulfide bond, we introduced C22L and C96A mutations into a published nanobody against GFP (GFPNB, PDB, 3K1K)^47^. Supplementary Fig. 2 shows that the disulfide bond-free GFPNB can still bind to EGFP to a substantial degree on the yeast cell surface. This is consistent with a previous study that GFPNB with these two cysteine residues mutated maintains the nanobody’s binding function^48^. Therefore, we incorporated C22L and C95A (corresponding to C96 in GFPNB) mutations to the synthetic nanobody scaffold to generate the disulfide bond-free nanobody libraries (Fig. 1a, Supplementary Fig. 3a). Without immunization, we constructed the synthetic nanobody libraries using degenerated primers to randomize CDR1, 2, and 3 following a recent protocol^42^ (detailed PCR construction in Supplementary S3 and Supplementary Method). The CDR3 was constructed with three different lengths with 7, 11, or 15 amino acids totally randomized with NNK degenerated codons to generate three libraries: DNA library 7, 11, and 15. The nanobody library size obtained for each library was ~1 × 10^7^, covering only part of all the possible amino acid combinations. These nanobody libraries were displayed on the yeast surface via fusion to the binding subunit of α-agglutinin protein Aga2p, a yeast surface protein (Fig. 1b) following an established protocol^49^.

### Selection of nanobodies against α-syn PFF

Next, we performed the selection of disulfide bond-free nanobodies against α-syn PFF. As illustrated in Fig. 1b, nanobodies were expressed on the yeast surface by fusion to the C-terminus of Aga2p, followed by the FLAG tag. α-Syn PFF was incubated with the yeast cell libraries to allow binding to nanobody clones. Then, mouse anti-α-syn monoclonal antibody (mAb) (BD Biosciences) was added to label α-syn PFF bound on the yeast cell surface, followed by the incubation with anti-mouse IgG conjugated with, either magnetic beads for magnetic-activated cell sorting (MACS) or fluorophores for fluorescence-activated cell sorting (FACS). By using MACS, nanobody clones binding with α-syn PFF were selected in the first round of sorting from a large population of yeast cells (~5 × 10^8^ cells). From the MACS enriched clones, we performed 5 more rounds of FACS and MACS (Fig. 1b) to enrich nanobody clones binding with α-syn PFF (Fig. 1c). Details of the selection process are described in Supplementary Fig. 3b, c.

From the enriched yeast cells, we extracted the plasmid DNA and re-transformed the DNA into bacterial cells for individual clone sequencing. From the 40 nanobody clones that were sequenced, 28 unique clones were identified (Supplementary Table 1). We re-transformed these nanobody clones into yeast cells for further characterization of the individual clones. Supplementary Fig. 4 shows that all these 28 clones showed preferential binding to α-syn PFF over α-syn monomers. Henceforward, we refer to these nanobodies as PFF-nanobodies (PFFNBs).

Because CDR3 is the antigen-binding loop with the most variations, we performed an amino acid analysis of loop3 for the selected PFFNBs. The majority of the selected clones consist of a CDR3 with 7 amino acids randomized (Supplementary Fig. 5a). The selected PFFNBs tend to be rich in hydrophobic and positively-charged residues in the CDR3 (Supplementary Fig. 5b). These PFFNBs may exhibit preferential binding to α-syn PFF through hydrophobic and ionic interactions.

### In vitro characterization and validation of the selected PFFNB2 binding to α-syn PFF

To validate the PFFNBs’ binding with α-syn PFF, we made PFFNB constructs fused with maltose binding protein (MBP) at its N-terminus for protein expression in *E. coli* (BL21). However, all of these nanobody proteins were retained in the cell pellet when expressed in *E. coli* (BL21) except for the positive control, GFPNB (C22L, C95A). This indicated that these PFFNBs are less stable than GFPNB (C22L, C95A) (Supplementary Fig. 6a). Successful expression of soluble PFFNBs was achieved by supplementing chaperon protein (plasmid pGro7) to BL21(C14) *E. coli* cells. Recombinant MBP-PFFNBs with a prominent band at the correct molecular weight were then observed in both the crude cell lysate and the semi-purified protein extract (Supplementary Fig. 6b) with polyacrylamide gel electrophoresis (PAGE) analysis. Supplementary Fig. 6c showed PAGE analysis of recombinant proteins which were later used in this study.

**Fig. 2:**
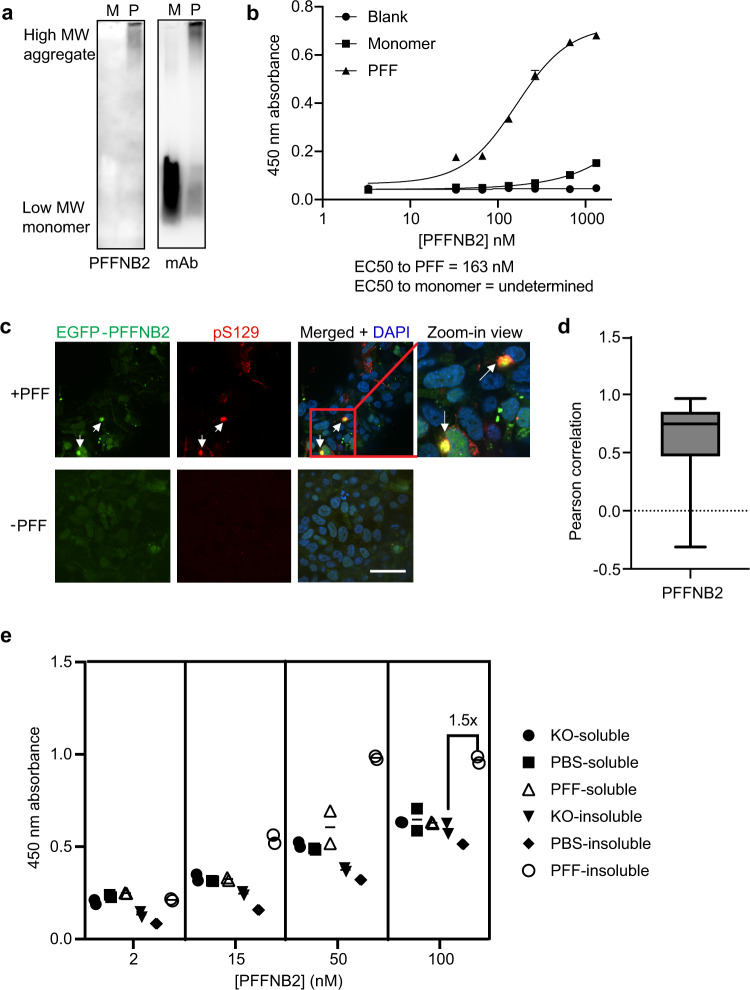
In vitro characterization of PFFNB2 binding to α-syn PFF and aggregates. **a** Native-PAGE immunoblot of human α-syn monomers and PFF with PFFNB2 and anti-α-syn monoclonal antibody (mAb). PFFNB2 binds selectively to the high molecular weight (MW) α-syn but not to the low MW α-syn. Anti-α-syn mAb binds to both the high and low MW α-syn forms. M, α-syn monomers. P, α-syn PFF. mAb, anti-α-syn monoclonal antibody. The experiment was replicated three times with similar results. **b** ELISA result of PFFNB2 binding to α-syn PFF, monomers, and control (blank). Wells were coated with 3 ng/μl of α-syn PFF or monomers, and then titrated with 3.3, 33.3, 66.7, 133.3, 266.7, 666.7, and, 1333.3 nM of PFFNB2. Three data points were collected for each concentration and shown as mean ± SEM. The experiment was replicated once with similar result. **c** AAV-transduced EGFP-PFFNB2 (green) signal co-localized with the immunostaining of anti-pS129 in HEK293T cells stably expressing α-syn(A53T) induced by α-syn PFF. Green, EGFP-PFFNB2 signal. Red, anti-pS129 immunofluorescence signal. White arrows indicated the co-localization between EGFP-PFFNB2 and pS129 α-syn. Scale bar, 40 μm. **d** Quantification of co-localization between pS129 α-syn signal to PFFNB2 using Pearson correlation. Data were analyzed from 103 puncta. The box ranges from the first to the third quartile of the distribution with median indicated as line across the box. The whiskers are the minimum and maxima of the data. **e** ELISA analysis of PFFNB2 binding to mouse brain lysate. KO, *Snca* knock-out mouse; PBS, transgenic mouse expressing human α-syn with striatal-PBS injection; PFF, transgenic mouse expressing human α-syn with striatal-PFF injection. Wells were coated with 3 ng/μl of each brain lysate, and then detected with 2, 15, 50, 100 nM of PFFNB2. Two data points were collected for each concentration. The experiment was replicated once with similar result. Source data are provided as a Source Data file.

We picked seven out of the 28 PFFNB clones for the initial testing. These nanobody clones were expressed, purified, and immunoblotted against α-syn PFF and monomers with native-PAGE. As published, anti-α-syn mAb (BD Biosciences) can detect total α-syn (monomers and aggregates) (Fig. 2a)^50^. Among the seven nanobody clones tested, MBP-PFFNB2 was identified to specifically bind α-syn aggregates, but not to α-syn monomers (Fig. 2a). Next, we performed ELISA to evaluate PFFNB2’s selective binding for α-syn PFF. MBP-PFFNB2 showed preferential binding to α-syn PFF (EC50, 163.0 nM) over α-syn monomers (EC50, undetermined) (Fig. 2b). This ELISA result is consistent with the result of native-PAGE immunoblot. To exclude any role of MBP in the binding, we further determined that MBP does not bind to α-syn PFF with ELISA (Supplementary Fig. 7a). Anti-α-syn mAb^50^ and MBP-NbSyn87^38^ exhibited comparable (non-selective) binding affinities toward α-syn PFF and monomers (Supplementary Fig. 7b, c). Further characterization with ELISA showed a similar binding affinity of MBP-PFFNB2 to human α-syn(A53T) PFF (EC50, 176.4 nM), a familial PD mutant^51^, compared to human wild type (WT) α-syn PFF (Supplementary Fig. 7d).

To evaluate the functional difference of PFFNB2 with or without disulfide-bond, we added back cysteines to residues 22 and 95. Re-introduction of the disulfide bond significantly reduced the binding affinity of PFFNB2 with α-syn PFF (Supplementary Fig. 7e). This suggests that disulfide bond may cause changes in the nanobody scaffold resulting in loss of function, which supports the rationale of generating disulfide bond-free nanobody libraries.

Next, we evaluated the expression and binding of PFFNB2 to α-syn PFF in the cytosol of mammalian cells. To study the interaction between α-syn aggregation and PFFNB2 in cells, we administered α-syn PFF to HEK293T cells co-expressing human α-syn(A53T) and EGFP-PFFNB2, following an established protocol^52^. Two days later, the cells were fixed and immunostained to assess the co-localization between EGFP-PFFNB2 and phosphorylated α-syn at serine129 (pS129). pS129 is a typical pathological α-syn marker^11,53^. Figure 2c showed that the pS129 level increased 2 days after α-syn PFF transduction, consistent with the previous study^52^. The puncta formed by the EGFP-PFFNB2 fusion protein (green) co-localized with the immunostaining of anti-pS129 (red puncta) (Fig. 2c, d). This result indicated that intracellularly-expressed PFFNB2 has the proper folding and function. More importantly, PFFNB2 not only binds to recombinant α-syn PFF, but also binds to the cellular inclusion of pS129-positive α-syn aggregates (Fig. 2c, d). We also attempted to perform the immunostaining of PFFNB2 in fixed cells containing α-syn aggregates (either with methanol fixation or paraformaldehyde fixation). However, there was no specific signal, probably because fixation could alter the conformational epitope of α-syn aggregates^54^. In brief, PFFNB2 can recognize pS129-positive α-syn aggregates in live cells, but not in fixed samples.

To further characterize the binding of PFFNB2 to α-syn pathology in brain samples, we prepared three types of brain samples: (1) α-syn transgenic mouse (PAC-Tg(SNCAWT), strain: 010710^12,55^) harboring *Snca* knockout allele, and a transgene encoding the human α-syn with intrastriatal injection of α-syn PFF, followed by 1-month incubation, which is PFF group; (2) PAC-Tg(SNCAWT) mice with striatal-PBS injection (PBS group); (3) *Snca* knockout (KO group) mice (C57BL/6-Snca^tm1MJMjff^/J, strain: 016123^55,56^). A total of six fractions were isolated (i.e., soluble and insoluble) from these three types of mouse brains using well-established protocols^12,44^. The pS129-positive α-syn pathology was first validated using immunoblot in the insoluble fraction of the PFF group, but not in the other five fractions (Supplementary Fig. 7f). Next, we performed ELISA on these brain samples with PFFNB2 at four different concentrations (2 nM, 15 nM, 50 nM and 100 nM). PFFNB2 exhibits higher binding to the insoluble fraction of the PFF group than the other five groups at concentrations of 15 nM, 50 nM and 100 nM (Fig. 2e). Taken together, these results showed that PFFNB2 can selectively bind to the native form of α-syn aggregates in mouse brain lysate.

### The dissociation of α-syn fibrils by PFFNB2 in vitro

Because PFFNB2 can specifically bind to α-syn PFF, we further evaluated whether PFFNB2 can affect α-syn aggregation. To determine whether PFFNB2 can inhibit α-syn aggregation, we performed an α-syn aggregation assay using 2 mg/ml recombinant α-syn monomers with 0.04 mg/ml (2%) recombinant MBP-PFFNB2 or MBP. MBP-PFFNB2 cannot significantly reduce the ThT signal in the α-syn aggregation assay compared to the MBP group, indicating that MBP-PFFNB2 cannot inhibit α-syn aggregation (Supplementary Fig. 8).

**Fig. 3:**
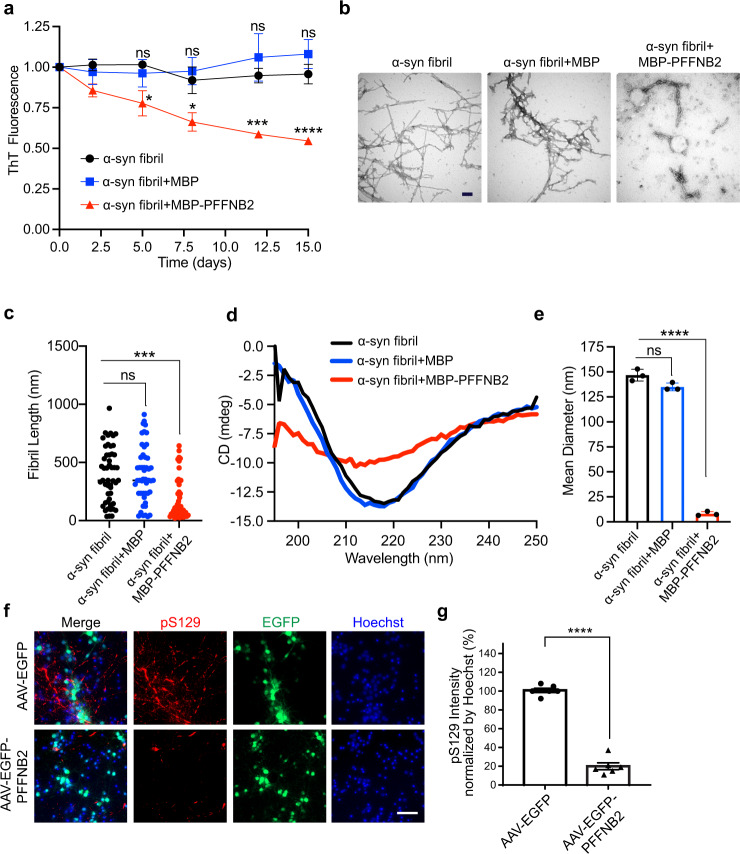
PFFNB2 dissociates α-syn fibrils and inhibits α-syn pathology induced by PFF in vitro. **a** Disaggregation of α-syn fibrils monitored by ThT fluorescence assay. α-syn PFF was incubated alone (black), with MBP-PFFNB2 (red), or with MBP (blue). Quantification data are the means ± SEM, *n* = 3 independent experimental replicates, two-way ANOVA with Tukey correction. (α-syn fibrils vs. α-syn fibrils + MBP-PFFNB2, *P* values are Day5 = 0.0388, Day8 = 0.0199, Day12 = 0.0005, Day15 = 0.0001) *P < 0.05, **P < 0.01, ***P < 0.001, ns, not significant. **b** TEM images of α-syn fibrils with MBP-PFFNB2 or MBP. Scale bar, 100 nm. **c** Quantification of fibril length from (**b**). Quantification data are the means ± SEM, *n* = 50 datapoints per group, one-way ANOVA with Tukey correction. (α-syn fibrils vs. α-syn fibrils+MBP-PFFNB2, *P* = 0.0001) ***P < 0.001, ns, not significant (**d**) Circular dichroism (CD) spectra for α-syn PFF with MBP-PFFNB2 or MBP. **e** Dynamic light scattering (DLS) analysis for α-syn fibrils with MBP-PFFNB2 or MBP. Quantification data are the means ± SEM, *n* = 3 independent experiments, *P* values were determined by one-way ANOVA with Tukey correction. (α-syn fibrils vs. α-syn fibrils+MBP-PFFNB2, *P* = 0.0001). ****P < 0.0001, ns, not significant. **f** WT mouse primary cortical neurons were transduced with AAV encoding EGFP (control group) or EGFP-PFFNB2 (PFFNB2 group) at day 5 in vitro and α-syn PFF at day 7 in vitro. The α-syn pathology level was assessed with anti-phosphorylated serine129 (pS129) immunostaining 7 days after α-syn PFF treatment. Scale bar, 50 μm. **g** Quantification of the pS129 immunoreactivity normalized by Hoechst. Quantification data are the means ± SEM, *n* = 6 independent experiments, *P* values were determined by two-sided Student’s *t* test. (AAV-EGFP vs. AAV-EGFP-PFFNB2 *P* = 0.0001). ****P < 0.0001. Source data are provided as a Source Data file.

We then sought to determine whether PFFNB2 can disaggregate α-syn fibrils. Mature α-syn fibrils were generated (the preparation step before sonication for α-syn PFF) as published^44^, and then incubated with MBP-PFFNB2 or MBP alone for 15 days (37 °C, 1000 r.p.m.). We assessed the level of α-syn aggregation using a ThT assay, and found that MBP-PFFNB2 can significantly reduce the ThT signal of α-syn fibrils, compared to the MBP group (Fig. 3a). There is no significant reduction of the ThT signal in the MBP group (Fig. 3a). We further assessed the morphology of the α-syn fibrils with TEM, and found that MBP-PFFNB2 can disrupt the fibrillar structures (Fig. 3b) with reduced fibril length (Fig. 3c). No appreciable change of fibril morphology or fibril length was observed in the MBP group (Fig. 3b, c).

Because fibrillar α-syn exhibits elevated β-sheet secondary structure^57,58^, we further determined whether MBP-PFFNB2 can reduce the β-sheet-enriched structure of α-syn fibrils with circular dichroism (CD) spectroscopy. Figure 3d showed the typical negative peak at 218 nm of the β-sheet secondary structure of α-syn fibrils. The addition of MBP-PFFNB2, but not MBP, reduced the negative ellipticity at 218 nm. Lastly, MBP-PFFNB2 significantly reduced the mean diameter of α-syn fibrils compared to MBP with dynamic light scattering (DLS) (Fig. 3e), which is consistent with the abovementioned ThT, TEM, and CD results. Taken together, our results showed that PFFNB2 can destabilize α-syn fibrils.

### AAV-EGFP-PFFNB2 decreased α-syn pathology in primary cortical neurons

Encouraged by the dissociation result, we further evaluated whether PFFNB2 can reduce α-syn pathology in WT mouse primary cortical neurons. AAV encoding EGFP-PFFNB2 (AAV-EGFP-PFFNB2) was added to cortical neurons at 5 days in vitro (DIV), followed by the administration of α-syn PFF at 7 DIV. AAV encoding EGFP (AAV-EGFP) was used as the control. Both substantial expression of EGFP in the AAV-EGFP and AAV-EGFP-PFFNB2 groups appeared at 11 DIV (Supplementary Figs. 9a, 8b). There was no difference in neurotoxicity 10 days after AAV transduction (neurons 15 DIV) between these two groups (Supplementary Fig. 9c, d). Substantial immunoreactivity of anti-pS129 in the AAV-EGFP group was observed in cortical neurons 7 days after α-syn PFF administration as published^10^. In contrast, AAV-EGFP-PFFNB2 induced a significant reduction of the immunoreactivity of anti-pS129 (Fig. 3f, g). Of note, in the experimental timeline, α-syn PFF was administered at 7 DIV, and EGFP-PFFNB2 expression was prominent at 11 DIV cortical neurons, 4 days after the PFF administration. This indicated that AAV-EGFP-PFFNB2 exhibited efficacy in inhibiting α-syn PFF-induced pathology in cortical neurons.

### AAV-EGFP-PFFNB2 prevented α-syn pathology spreading to the cortex induced by intrastriatal injection of α-syn PFF in vivo

**Fig. 4:**
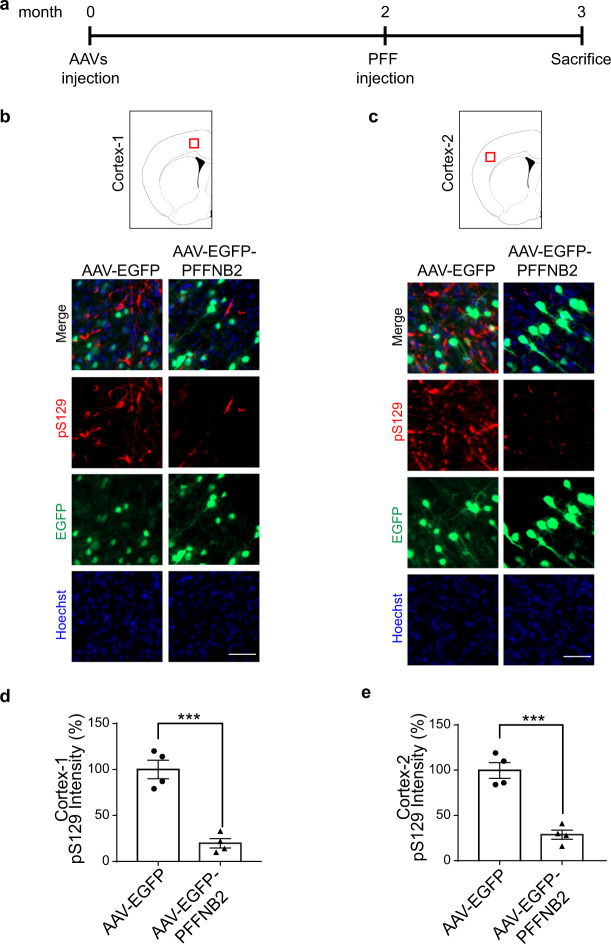
AAV-EGFP-PFFNB2 reduces α-syn pathology induced by PFF in vivo. **a** Timeline of in vivo experiments. Intraventricular injection of AAV was performed in neonatal mouse brains. These mice were then stereotactically injected with α-syn PFF at 2-months old, and then sacrificed 1 month after α-syn PFF injection. **b**, **c** Immunostaining of pS129 (red) normalized by Hoechst (blue). The green signal indicates the expression level of EGFP or EGFP-PFFNB2 fusion protein in the cortex. Scale bars, 50 μm. **d**, **e** Quantification of pS129 immunostaining in the cortex. Data are the means ± SEM, *n* = 4 mice per group, *P* values were determined by two-sided Student’s *t* test. (AAV-EGFP vs. AAV-EGFP-PFFNB2, *P* = 0.0004). ***P < 0.001. Source data are provided as a Source Data file.

To test the efficacy of PFFNB2 in mediating human α-syn pathology in vivo, we chose the α-syn transgenic mouse (PAC-Tg(SNCAWT), strain: 010710^12,55^) lacking endogenous mouse α-syn but expressing human α-syn. We performed intraventricular injection of AAV encoding either EGFP-PFFNB2 or EGFP to these neonatal mice following the well-established protocol^59^. Two months after AAV injection, intrastriatal injection of α-syn PFF was performed on these mice when in the adult age, to induce α-syn pathology spreading to the cortex that was examined one month later^11^ (Fig. 4a).

**Fig. 5:**
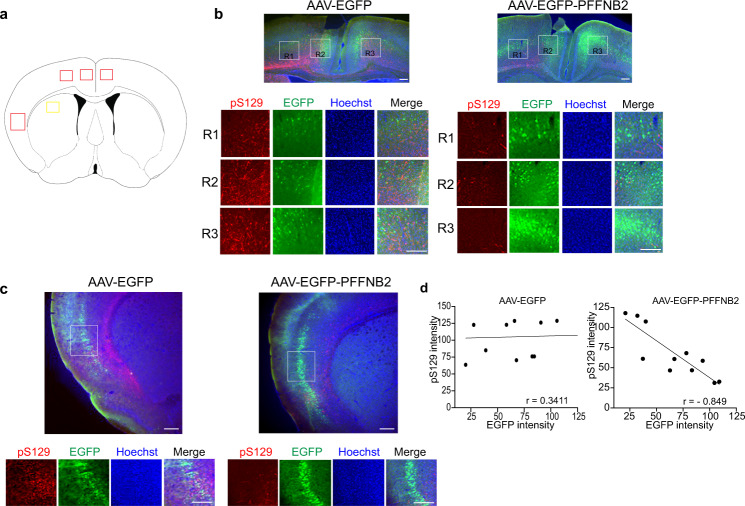
Lower magnification of striatal-PFF injected mice brain section with AAV-EGFP and AAV-EGFP-PFFNB2 transduction. Both EGFP and EGFP-PFFNB2 expression (green) were mainly expressed in the motor cortex and somatosensory cortical regions. **a** Schematics representing the PFF injection site (yellow box) and site of analyses (red box) (**b**) The ×4 magnification images of the motor cortical region (top panel) and marked regions R1, R2, and R3 (white boxes) are shown in the bottom panel. Scale bars, 100 μm. **c** The ×4 magnification images of the somatosensory cortical region (top panel) and marked area (white box) is shown in the bottom panel. All experiments were replicated in three mice per group with similar results. Scale bars, 100 μm. **d** Correlation between EGFP intensity to pS129 intensity plot in the AAV-EGFP and AAV-EGFP-PFFNB2 groups. Areas with low to high degrees of EGFP expression were selected for quantification. There is a negative correlation between EGFP intensity and pS129 intensity in the AAV-EGFP-PFFNB2 group, with the Pearson correlation coefficient *r* = −0.8490. There is no correlation between EGFP and pS129 in the AAV-EGFP group with *r* = 0.3414. Source data are provided as a Source Data file.

One month post-striatal-PFF injection, we assessed the EGFP expression in the cortex, and the immunoreactivity of anti-pS129 in the cortex of these mice. The intraventricular injection of the AAV with synapsin promoter resulted in the expression of EGFP in the neurons of the cerebral cortex (Figs. 4a, c, 5a–c), consistent with the published study^59^. The EGFP was mainly expressed in the motor and somatosensory cortical regions (~12–18%) (Fig. 5b, c). Two cortex sub-regions with high intensity expression of EGFP were chosen for analysis. Substantial immunoreactivity of anti-pS129 was observed in the AAV-EGFP group (Figs. 4b–e, 5a–c), indicating that α-syn pathology spread from the striatum to the cortex. In contrast, a significant decrease of pS129 level was detected in the AAV-EGFP-PFFNB2 group (Figs. 4b–e, 5a–c). Furthermore, we performed the correlation analysis between the intensity of EGFP expression and pS129 immunoreactivity. The results showed that pS129 immunoreactivity is negatively correlated with the intensity of EGFP in the AAV-EGFP-PFFNB2 group, and there is no relevant correlation in the AAV-EGFP group (Fig. 5d). In another, there is no significant reduction of pS129 level in the striatum of the AAV-EGFP-PFFNB2 group, which could be attributed to the absence of EGFP-PFFNB2 expression in the striatum (Supplementary Fig. 10a, b). Taken together, AAV-EGFP-PFFNB2 can effectively prevent pathogenic α-syn spreading to the cortex in the striatal-PFF mice model.

## Discussion

Fibrillar α-syn aggregates are prion-like seeds propagating throughout the brain, which is a major driver in the pathogenesis of LBD, PD, and related α-synucleinopathies^7–9^. It is important and necessary to generate reagents that specifically target α-syn fibrils. To address this, we establish an extracellular disulfide bond-free nanobody selection platform that allows the generation of nanobodies with consistent folding and function in both intracellular and extracellular environments. We generated and identified a nanobody (PFFNB2) that specifically binds to α-syn PFF, but not to α-syn monomers. PFFNB2 dissociated α-syn fibrils in the solution, inhibited α-syn pathology in vitro, and prevented α-syn pathology from spreading to the cortex by AAV-transduction. We expect that these PFFNB-related agents hold great promise as a potential therapeutic strategy against α-syn-related pathogenesis.

Native α-syn monomers play an important role in vesicle trafficking and refilling at synapses^60–62^. Thus, targeting α-syn monomers may result in the imbalance between the reserved and releasable vesicles, and impair neurotransmitter uptake by the vesicles^62,63^. Non-conformationspecific reagents (e.g., antibody, nanobody) might also disrupt the balance of α-syn monomers and tetramer^64,65^, and α-syn tetramer has been reported to inhibit α-syn aggregation. Of note, α-syn fibrils are more toxic than other species^66^ (e.g., oligomer, ribbon). Thus, targeting α-syn fibrils is critical for therapeutic development against LBD, PD, and related α-synucleinopathies.

Considering the observation of cellular α-syn pathological inclusions and abundant expression of cytosolic α-syn monomers in neurons, it is critical to block the intracellular propagation of endogenous α-syn induced by the internalized α-syn seeds. The efficacy of α-syn fibril-specific antibodies in reducing α-syn pathology has been reported^27^. However, antibodies have poor membrane penetration ability and are usually not functional in the reducing cytosolic environment^21,22^, and therefore, can only target extracellular α-syn fibrils^23–27,67^, limiting their impact on the pre-existing intracellular α-syn aggregates. A study has reported that single-chain antibody fragment (scFv) D5 binding to α-syn oligomer can inhibit α-syn fibrillization^68^ and toxicity^69^. However, intracellular expression of scFV tends to aggregate, which could compromise its therapeutic effects^35^. Overall, nanobodies are preferred over conventional antibodies or scFVs for intracellular applications, as they are small, monomeric, stable, and commonly expressed inside cells^31^. We believe this work provides a proof-of-concept that targets intracellular α-syn aggregates using AAV transduced α-syn-fibril-specific nanobody can effectively reduce pathology propagation.

In this study, we focus on using AAV-PFFNB2 to target intracellular α-syn aggregates against pathogenic α-syn spreading. Of note, emerging evidence also suggests the importance of exogenous α-syn aggregates in α-syn cell-to-cell transmission, by entering neurons and seeding endogenous α-syn aggregation^12,70,71^. Encouragingly, two α-syn antibodies, PRX002^23,72,73^ and MEDI1341^74^ that bind to total α-syn, have exhibited efficacy in ameliorating  the disease phenotype of PD models when administered extracellularly, and they are currently in Phase II (ID: NCT03100149) and Phase I (ID: NCT04449484) clinical trial, respectively. More study is needed to evaluate the efficacy of exogenously applied PFFNB2 in preventing the neuronal uptake of α-syn PFF and subsequent α-syn propagation. If recombinant PFFNB2 can inhibit the effects of exogenous α-syn PFF when administered extracellularly, we will then modify the AAV-PFFNB2 system to secret PFFNB2 to the extracellular space. PFFNB2 could also be expressed in a bacteria-based expression system, which is higher-yielding and more cost-effective than the eukaryotic cell-based system used for conventional antibody production^75^. Further bioengineering efforts on PFFNB2 will also be necessary to improve brain penetration^76^.

Because the conserved cysteine residues will not form a disulfide bond in the reducing environment of the bacterial cytoplasm during the selection process, an intracellular bacterial selection platform could be advantageous for selecting nanobodies without forming a disulfide bond^77^. However, an extracellular nanobody selection platform such as yeast surface display is still needed, for selecting nanobodies against targets that can only be supplemented extracellularly, such as α-syn PFF. In the extracellular nanobody selection platform, the conserved cysteine residues would form a disulfide bond during the selection process. Therefore, in our library design, we introduced cysteine mutations to remove the disulfide bond even in the extracellular oxidizing condition. Although a previous study^48,77^ showed that removing the conserved disulfide bond in nanobodies does not have a significant effect on their binding affinity in the reducing environment, our study suggests that the disulfide bond can change the binding property of a nanobody. Thus, it is important to generate a nanobody framework exhibiting consistent binding property from the selection stage to final applications.

In summary, we have demonstrated the feasibility of selecting disulfide bond-free nanobodies that can be stably expressed in the cytoplasm to target the α-syn fibrillar form over the monomeric form. As a research tool, expressing EGFP-PFFNB2 in specific brain regions of mouse models with α-syn pathology propagation would enable interrogation of their functions in disease progression. PFFNB2 could be utilized to study pathology development in different brain regions and their effects on the alleviation of behavioral and cognitive deficits. Lastly, with further investigation and development, PFFNB2 holds therapeutic promise for treating LBD, PD, and related α-synucleinopathies.

## Methods

This research complies with all relevant ethical regulations. The animal studies were approved by Johns Hopkins University Animal Care and Use Committee (ACUC). All animal studies were performed according to the NIH Guide for the Care and Use of Experimental Animals and the guidelines of the Institutional Animal Care Committee of the Johns Hopkins University.

### α-Syn PFF generation

Recombinant human WT and A53T α-syn monomers were purified following the established protocol^44^. Briefly, Full length human WT or A53T α-syn containing pRK172 vector were expressed in BL21(DE3) *E. coli*. α-syn monomers were subsequently purified using Hi-Trap Q HP anion exchange column (GE Healthcare Life Sciences). To obtain α-syn PFF, the purified α-syn monomers were diluted in phosphate buffer saline (PBS) (5 mg/ml) followed by agitation for 7 days (1000 r.p.m., 37 °C) to prepare the mature fibrils. Then the aggregates were collected by centrifugation and resuspended with endotoxin-free PBS. The aggregates were sonicated for 1 min into PFF (1 s on, 1 s off, 30% amplitude) with a sonicator (Branson Ultrasonics). The concentration of α-syn was determined by BCA assay (Thermo Fisher Scientific). To confirm aggregate formation, recombinant α-syn monomers or PFF were diluted in PBS buffer (100 μg/ml) followed by incubation with 50 μM Thioflavin T (ThT). The ThT fluorescence was measured using a plate reader (Varioskan LUX plate reader, Thermo Fisher Scientific) with excitation at 450 nm and emission at 485 nm. To perform TEM analysis, a drop of 30 μl α-syn monomers or PFF sample (100 ng/μl) were loaded onto carbon-coated 400 mesh copper grids (Electron Microscopy Sciences). The grids were washed with 30 μl water three times and then negatively stained with 2% uranyl formate. The excess buffer was removed by filter paper. The images were acquired with Philips/FEI BioTwin CM120 Transmission Electron Microscope.

### Nanobody library generation

Nanobody libraries were constructed by overlapping polymerase chain reaction (PCR), combining a series of degenerated primers to generate the DNA encoding full-length nanobody. Degenerated bases were used to randomize CDR1, 2, and 3. Three different lengths were constructed for CDR3 to generate Library 7, 11, and 15 (the number corresponds to the number of amino acids randomized on CDR3). To enable homologous recombination of these randomized nanobody DNA constructs into the yeast vector backbone, these DNA constructs are flanked with 42 base pairs that are overlapping with the pCTCON2 vector backbone. The pCTCON2 vector was previously engineered to contain Aga2p and FLAG-tag with NheI and BamHI cut sites in between for nanobody gene insertion. After homologous recombination, nanobodies were expressed on yeast surface with FLAG-tag at its C-terminus to monitor nanobody expression. Details of construction with primer sequences and the sequence of nanobody libraries could be found in Supplementary Table 3 and 4. A detailed description of the nanobody library construction using overlapping PCR is included in the Supplementary Method.

After preparation of the linearized vector and randomized nanobody PCR fragments, we followed the established protocol for yeast competent cell preparation and electroporation^78^. Briefly, EBY100 yeast competent cells (Thermo Fisher Scientific) were pre-conditioned with 100 mM lithium acetate and 1 mM dithiothreitol and then electroporated with a mix of the nanobody PCR fragments and linearized vector. Electroporated yeast cells were grown in SDCAA media (synthetic dextrose plus casein amino acid, 2% dextrose, 0.67% yeast nitrogen base without amino acids (BioBasic), 0.5% Bacto casamino acids (Difco), 0.54% disodium phosphate, 0.856% monosodium phosphate) lacking tryptophan as a selection marker at 30 °C and 200 r.p.m. for 24 h. The library size of the nanobody libraries generated was ~1 × 10^7^ for each library.

### PFFNB selection and DNA sequence analysis

Roughly 1.5 × 10^8^ yeast cells from each library (Library 7, 11, and 15) were cultured and protein expression was induced with 1:10 dilution of SDCAA in SGCAA media (synthetic galactose plus casein amino acid, 2% galactose, 0.67% yeast nitrogen base without amino acids (BioBasic), 0.5% casamino acids (Difco), 0.54% disodium phosphate, 0.856% monosodium phosphate) overnight at 30 °C. The next day, the yeast cells grew up to 10^8^ cells/ml. About 5 × 10^8^ cells were pelleted to be incubated with α-syn PFF for 1 h at room temperature (RT). Following that, the yeast cells were immunostained with mouse anti-α-syn mAb (1:200 dilution, BD Biosciences, Cat no. 610787) for 1 h at RT. For MACS, yeast cells were kept in MACS buffer (PBS pH 7.2, 0.5% Bovine Serum Albumin, 2 mM EDTA). Then cells were immunostained first with goat anti-mouse-AlexaFluor 647 antibody (1:400, Thermo Fisher Scientific, Cat no. A21235) and then anti-Cy5-microbeads (20 uL/10^7^ cells, Miltenyi Biotech, Cat no. 130-042-401), each for 30 min at RT. After immunostaining, yeast cells were loaded onto the LS column (Miltenyi Biotech) followed by 3× washing with MACS buffer. LS column was then removed from the magnetic apparatus and flushed with MACS buffer to elute the bound cells.

For FACS, the yeast cells were first incubated with PFF, followed by primary antibodies mouse anti-α-syn mAb (1:200 dilution, BD Biosciences, Cat no. 610787) and rabbit anti-FLAG antibody (1:200 dilution, Sigma, Cat no. F7425) and then secondary antibodies goat anti-mouse IgG-AlexaFluor 568 antibody (1:400 dilution, Thermo Fisher Scientific, Cat no. A11004) and goat anti-rabbit IgG-AlexaFluor 647 antibody (1:400 dilution, Thermo Fisher Scientific, A21245). FACS sorting was performed using BD FACSAria III cell sorter and BD FACSDiva software (BD Biosciences). Analysis of the FACS plot was performed using FlowJo software. After several rounds of alternating MACS and FACS, the nanobody DNA plasmids were isolated and re-transformed to XL1-Blue *E. coli* competent cells. Forty PFFNB clones were sequenced and analyzed with Sanger sequencing.

### Yeast re-transformation

From the 40 PFFNB clones sequenced, 28 unique clones were identified and re-transformed into yeast chemical competent cells individually using Frozen-EZ yeast Transformation Kit (Zymo Research). Each of these nanobody clones was labelled with α-syn PFF or monomers and then immunostained and analyzed using BD FACSAria III (BD Biosciences) as described above in PFFNB selection.

### PFFNB protein expression and purification

The PFFNBs were cloned into the pYFJ16 plasmid for protein expression and purification (listed in Supplementary Table 4). For Native-PAGE gel immunoblotting experiment, ELISA and dissociation assay, HisTag-MBP-FLAG, MBP-PFFNB2-FLAG, MBP-PFFNB2(L22C, A95C)-FLAG, and MBP-NbSyn87-FLAG were used (see Supplementary Table 3 for details). The PFFNB clones were transformed into BL21 *E. Coli* (C14) (gift from Dr. Haoming Zhang, University of Michigan) expressing a set of chaperones from plasmid pGro7 (Takara Bio). The transformed BL21 cells were grown in 5 ml Luria Broth (LB) media overnight at 37 °C and 220 r.p.m. The next morning, the 5 mL bacterial cultures were transferred to 500 ml LB and grown until OD_600_ = 3. Then, 1 mM IPTG was added to cells to induce protein expression. Cells were shaken at 220 r.p.m. and 16 °C for 16 h.

Cells were pelleted and lysed using B-PER Bacterial Protein Extraction Reagent (Thermo Fisher Scientific). The soluble proteins in the cell lysates were extracted first with Ni-NTA resin (New England Biolabs) and then size exclusion gel chromatography (AKTA Go, Cytiva). The purified proteins were analyzed with SDS-PAGE.

### Mouse brain lysis and fractionation

Soluble and insoluble fractions of brain lysates were prepared as previously described^50^. Briefly, brain tissue was homogenized in soluble lysis buffer (10 mM Tris-HCl, pH 7.4, 150 mM NaCl, 5 mM EDTA, 0.5% Nonidet P-40, complete protease inhibitor cocktail, and Phosphatase Inhibitor). Tissue homogenate was centrifuged at 20,000 *g* for 30 min and the supernatant (soluble fraction) was collected and stored. The pellet was washed with soluble lysis buffer and the resulting pellet was resuspended into the insoluble lysis buffer (10 mM Tris-HCl, pH 7.4, 150 mM NaCl, 5 mM EDTA, 0.5% Nonidet P-40, complete protease inhibitor cocktail, and Phosphatase Inhibitor) containing 1% SDS and 0.5% sodium deoxycholate. The homogenate was sonicated for 10 s (2 min on, 3 min off) and centrifuged at 20,000 *g* for 30 min to collect the insoluble fraction.

### Immunoblot analysis

Samples were separated on Native-PAGE^™^ Bis-Tris Gels (Thermo Fisher Scientific) and transferred to PVDF membranes (Bio-Rad). The membranes were blocked with 5% BSA in TBST (Tris-buffered Saline-Tween 20) overnight at 4 °C. Then the membranes were incubated with purified MBP-PFFNB2-FLAG (~4 μg/ml) or mouse anti-α-syn mAb (1:2000 dilution, BD Biosciences, Cat no. 610787) in TBST with 5% Bovine Serum Albumin overnight at 4 °C. Mouse-anti-Flag-HRP antibody (1:5000 dilution, Sigma-Aldrich, Cat no. A8592) or anti-mouse IgG-HRP (1:5000 dilution, GE Healthcare, Cat no. NA931) were used as secondary antibody followed by incubation with SuperSignal West Pico Plus chemiluminescent substrate (Thermo Fisher Scientific). The images were acquired and processed with ImageQuant LAS 4000mini scanner (GE Healthcare Life Sciences) and Amersham Image 600 (GE Healthcare Life Sciences).

The soluble and insoluble fractions of brain lysates were resolved on 15% Tris-glycine gel and transferred to PVDF membranes for analysis with rabbit anti-α-syn (1:1000 dilution, Cell signaling, Cat#4179), rabbit anti-α-syn (pS129) (dilution 1:1000, Abcam, Cat no. ab51253) and mouse anti-ß-actin peroxidase (1:10,000 dilution, Millipore Sigma, Cat no. A3854) antibodies.

### ELISA

To determine the EC50 of PFFNB2 (in Fig. 2b), 300 ng of α-syn PFF or monomers were diluted in 100 μl of coating buffer (0.2 M sodium carbonate buffer pH 9.4). α-Syn PFF or monomers were plated on 96-well plates (Greiner Bio-One) (100 μl/well) followed by shaking at 200 r.p.m. at 4 °C overnight. Wells incubated with coating buffer served as the blank. The next day, all treatments were done at RT, with 200 r.p.m. shaking on a microplate shaker. First, each well was decanted and washed with 200 μl washing buffer (25 mM Tris, 150 mM NaCl, Tween 0.05%, pH 7.2) for 3 min. The washing step was repeated three times. Then the wells were blocked with SuperBlock blocking buffer containing 0.05% Tween-20 (Thermo Fisher Scientific) for 30 min, followed by incubation with purified recombinant MBP-PFFNB2-FLAG protein in SuperBlock at concentrations of 3.3, 33.3, 66.7, 133.3, 266.7, 666.7, and 1333.3 nM. The wells were then washed three times before incubation with mouse-anti-FLAG antibody (Sigma-Aldrich, 1:1000 dilution, Cat no. F3165) in SuperBlock for 1 h. Lastly, the wells were washed and incubated with Goat anti-mouse-IgG-HRP antibody (Thermo Fisher Scientific, 1:5000 dilution, Cat no. 31430) in SuperBlock for 1 h. 100 μl of TMB substrate (Thermo Fisher Scientific) were added into the wells and incubated for 15–30 min until blue color appeared. The reaction was stopped with an ELISA stop solution (Thermo Fisher Scientific). The absorbance of processed TMB substrate at 450 nm was quantified with a microplate reader (BioTek Cytation 5) and Gen5 software. Similar procedures were applied to acquire data in Fig. 2e, Supplementary Fig. 7a–e.

For Fig. 2e, 3 ng/μl of soluble or insoluble mouse brain lysates were used to coat the wells. For Supplementary Fig. 7a, 3 ng/μl of α-syn PFF were used to coat the wells then MBP-PFFNB2-FLAG and MBP-FLAG (as control) were used for titration. For Supplementary Fig. 7b, a similar ELISA protocol was used, except that α-syn PFF and monomers were plated on white opaque 96 well-plates (Costar) and the signal was detected using SuperSignal ELISA Pico chemiluminescent substrate (Thermo Fisher Scientific). For Supplementary Fig. 7c, purified MBP-NbSyn87-FLAG was used for titration of α-syn PFF and monomers. For Supplementary Fig. 7d, 3 ng/μl of recombinant human α-syn PFF with and without A53T mutation were used for coating the plates. For Supplementary Fig. 7e, MBP-PFFNB2-FLAG and MBP-PFFNB2 (L22C, A95C) were used for titration.

### HEK293T cell culture

HEK 293T/17 cell line (ATCC, cat#: CRL-11268) were used in this study and tested every 3 months for mycoplasma contamination by DNA staining. Cell morphology was also visually checked and the doubling rate was quantified (~24 h). No misidentified cell line was used in this study. Cells (<20 passages) were cultured at 37 °C under 5% CO_2_ in complete growth media (1:1 DMEM (Dulbecco’s Modified Eagle Medium, Gibco): MEM (Eagle’s Minimal Essential Medium) supplemented with 10% FBS (Fetal Bovine Serum, Sigma), 50 mM HEPES (Gibco), and 1% Penicillin-Streptomycin (Gibco). All plates and flasks used for cell culture were pre-treated with 20 µg/mL human fibronectin (Millipore Sigma) for 10 min at 37 °C.

### Production of lentiviruses

For the HEK293T cell experiment in Fig. 2c, d, the EGFP-PFFNB2 fusion gene was cloned into a modified pLX208 lentiviral vector that does not have a hygromycin selection marker (Supplementary Table 4). For generating α-syn stable cell line, α-syn(A53T) DNA (Integrated DNA Technologies) was cloned into the pLX208 plasmid with a hygromycin selection marker. 2.5 µg of these lentiviral DNA were then mixed with 0.25 µg pVSVG and 2.25 µg ∆8.9 lentiviral helper plasmid in 250 µl of DMEM. Then, the DNA mix was incubated with 25 µL PEI max solution (1 mg/ml, Polysciences) at RT for at least 10 min. The DNA and PEI mix was finally added to 70–90% confluent HEK293T cells in the T25 flask. After incubation at 37 °C for 36–48 h to allow lentivirus production, the supernatant with lentiviruses was collected for either immediate use or was flash frozen in liquid nitrogen and stored at −80 °C for future use.

### α-Syn(A53T)-HEK293T stable cell line generation

HEK293T cells were infected with lentiviruses expressing α-syn(A53T) monomers and selected with 150 µg/ml hygromycin (Thermo Fisher Scientific). α-syn(A53T)-expressing stable cell line was validated by immunostaining using mouse anti-α-syn mAb (1:2000 dilution, BD Biosciences, Cat no. 610787) and goat anti-mouse-AlexaFluor 568 (1:2000 dilution, Thermo Fisher Scientific, Cat no. A11004).

### HEK293T cell lentiviral infection and α-syn PFF transduction in α-syn(A53T)-stable cell line

For confirmation of PFFNBs binding to PFF in mammalian cells, HEK293T cells stably expressing α-syn(A53T) were plated on 24-well glass-bottom plates at 40–60% confluence. After 2 h incubation at 37 °C under 5% CO_2_ in complete growth media, the cells were then transduced with 10 ng recombinant human α-syn PFF using BioPorter (Genlantis). Three hours after transduction, 100–200 µl of each supernatant lentiviruses encoding EGFP-PFFNB2 was added to the cells. Cells were incubated for two more days before immunostaining.

### Immunostaining and immunofluorescence analysis of α-syn PFF-transduced HEK293T cells

Two days after transduction with or without α-syn PFF, HEK293T cells expressing EGFP-PFFNBs were fixed with 4% paraformaldehyde. The fixed cells were permeabilized using 0.1% Triton-X, followed by blocking with 5% BSA in PBS. Cells were stained with rabbit anti-p129S primary antibody (1:1000 dilution, Abcam, Cat no. ab51253) and DAPI (1:1,000, Bio-Rad, Cat no. 1351303) for 1 h at RT. The secondary stainings were performed with anti-rabbit IgG-AlexaFluor 568 (1:2000 dilution, Thermo Fisher Scientific, Cat no. A11036) for 1 h at RT.

Confocal imaging was performed on a Nikon Eclipse Ti2 inverted confocal microscope with 60X oil-immersion objectives, outfitted with Yokogawa CSU-X1 5000RPM spinning disk confocal head and ORCA-Flash 4.0 LT + sCMOS camera. The following combinations of laser excitation and emission filters were used for corresponding fluorophores: DAPI (405 nm excitation; 455/50 emission), EGFP (488 nm excitation; 525/36 emission), Alexa Fluor 568 (568 nm excitation; 605/52 emission). Region with high pS129 signal were chosen as region of interest (ROI), then the Pearson correlation analysis was performed using Nikon NIS-Elements software.

### Preparation of concentrated AAV with AAV1/2 mixed serotype

For the neuron culture infection and animal experiments, EGFP and EGFP-PFFNB2 fusion genes were cloned into AAV2 vector under synapsin promoter (Supplementary Table 4). To produce AAV, 3× T150 flasks of HEK293T cells (<20 passages, 100% confluency) were each transfected with 5.2 μg AAV vector, 4.35 μg AAV1, 4.35 μg AAV2 serotype plasmids, and 10.4 μg pDF6 adenovirus helper plasmid using PEI max. After 36–48 h incubation at 37 °C under 5% CO_2_, cells were collected with 10 mL DPBS and centrifuged at 30 *g* at RT for 5 min. The pellet was resuspended in 20 mL 100 mM NaCl, 20 mM Tris (pH = 8.0) and lysed with 1 mL freshly prepared 10% sodium deoxycholate (Sigma). Benzonase nuclease (Sigma) was added to a final concentration of 50 units per mL and the solution was incubated at 37 °C for 1 h with gentle agitation followed by centrifugation at 11,000 *g* for 10 min.

A heparin column (GE Healthcare) was first equilibrated with 10 ml of 100 mM sodium chloride (NaCl), 20 mM Tris (pH = 8.0) using a peristaltic pump. The viral supernatant was then loaded to the column followed by serial washing steps with 20 ml of 100 mM NaCl, 20 mM Tris (pH 8.0), 1 ml of 200 mM NaCl, 20 mM Tris (pH 8.0) and 1 ml of 300 mM NaCl, 20 mM Tris (pH 8.0). The AAVs were eluted sequentially with 1.5 ml 400 mM NaCl, 20 mM Tris (pH 8.0); 3.0 mL 450 mM NaCl, 20 mM Tris (pH 8.0) and 1.5 ml 500 mM NaCl, 20 mM Tris (pH 8.0). The eluted virus was concentrated using Amicon Ultra centrifugal units (Sigma) with a 100,000 Da cut off and buffer exchanged into sterile 20 mM Tris, 150 mM NaCl, 0.05% PF68 solution to a final volume of ~100 μl. The concentrated AAV was stored at −80 °C. AAV titer was measured with qPCR.

### Primary cortical neuron culture

Before primary cortical neuron culture, tissue culture plates were coated with Poly-L-Ornithine and washed three times with autoclaved milli-Q water. Primary cortical neurons were cultured from *embryonic* 15.5 days pups of CD-1 pregnant mice (Charles River) in Neurobasal Medium with B-27 Supplement (Thermo Fisher Scientific).

AAVs with titer 3.6 × 10^10^/μl were applied to primary cortical neurons on day 5. Then, α-syn PFF were treated into neurons on day 7 followed by incubation for 7 days before α-syn pathology assay. To check neurotoxicity, primary cortical neurons were transduced with AAVs with titer 3.6 × 10^10^/μl on day 5 and incubated further for 10 days before immunostaining with anti-NeuN antibody (Millipore-Sigma, MAB377).

### Animals

PAC-Tg(SNCAWT) mice (Strain: 010710) and C57BL/6-Snca^tm1MJMjff^/J mice (strain: 016123) were obtained from the Jackson Laboratory. Pregnant CD-1 mice (Strain: 022) were used for primary cortical neuron cultures (Charles River). All mice were housed under standard condition of constant temperature of (22 ± 1 °C), relative humidity of 42%, and 12 h light cycle with food and water. These animal studies were approved by Johns Hopkins University ACUC. All animal studies were performed according to the NIH Guide for the Care and Use of Experimental Animals and the guidelines of the Institutional Animal Care Committee of the Johns Hopkins University.

### Intraventricular injection of AAV

Intraventricular injection of AAV was performed as soon as the pups are nursing. The pups were transferred from the warming pad to the cold metal plate to induce hypothermia anesthesia. Injection sites were marked at 2/5 of the distance from the lambda suture to each eye then the needle was inserted to a depth of 3 mm. One μl of AAV1/2 mixed serotype AAV with titer 3.6 × 10^10^/μl were delivered into both hemispheres. After completing injections, the pups were placed back on the warming pad and monitored for wound healing and recovery after surgery.

### Stereotaxic injection

Two-month-old PAC-Tg(SNCAWT) mice (Jackson Laboratory) were anesthetized with a mixture of ketamine and xylazine before injection. α-Syn PFF (5 μg for each mouse) were briefly sonicated and stereotactically injected into the striatum at a rate of 0.2 μl per minute with the following coordinates: +2.0 mm medial-lateral; +0.2 mm antero-posterior; +2.6 mm dorsoventral from bregma. The needle was maintained in place for another 5 min after injection, and then slowly removed from the brain. After surgery, the mice were monitored for wound healing and recovery. For histological studies, the mice were sacrificed one month after injection followed by perfusion with ice-cold PBS and 4% paraformaldehyde. Mouse brains were fixed in 4% paraformaldehyde overnight and then transferred to 30% sucrose in PBS. A series of 40 μm coronal sections were prepared for immunostaining.

### Immunofluorescence analysis of α-syn PFF-treated primary cortical neuron culture and α-syn PFF-injected brain section

Primary cortical neurons were fixed with 4% paraformaldehyde followed by permeabilization with 0.2% Triton X-100. The cells were incubated overnight at 4 °C with rabbit anti-α-syn (pS129) antibody (dilution 1:1,000, Abcam, Cat no. ab51253) as primary antibody for α-syn pathology assay or mouse anti-NeuN antibody (dilution1:500, Millipore-sigma, Cat no. MAB377) for neurotoxicity, then incubation with anti-rabbit IgG secondary antibodies conjugated to Alexa-fluor 568 (1:1000 dilution, Thermo Fisher Scientific, Cat no. A11036) and Hoechst (1:5000 dilution, Thermo Fisher Scientific, Cat no. 62249) for 1 h at RT. For brain sections, 40 μm thick brain sections were blocked in with 10% goat serum with 0.3% Triton X-100 for 1 h at RT and then immunostained with the same method as culture studies. All images for primary cortical neuron culture and brain sections were taken with Zeiss Axio Observer Z1 then analyzed with Zen Lite software and ImageJ.

### α-Syn fibrils dissociation assay and transmission electron microscopy (TEM)

α-Syn fibrils were first generated by incubation of 2 mg/ml α-syn in PBS, pH 7.4 at 37 °C for 7 days at 1000 r.p.m. For in vitro dissociation assay, 5 μM of α-syn fibrils were incubated with 250 nM MBP or MBP-PFFNB2 for up to 15 days at 37 °C at 1000 r.p.m. The reaction mixture was taken out from an incubation solution at a different time interval (0, 2, 4, 8, 12, 15 days). 20 μM final concentration of ThT was added into clear bottom 96 well microplate (Invitrogen, Cat no.M33089), and fluorescence intensity was measured at 450 nm excitation and 485 nm emission wavelength using a Varioskan lux (Thermo scientific) multimode microplate reader. All measurements were performed in triplicate. All ThT fluorescence results were normalized with the ThT signal at zero time. The morphological analysis of PFFNB2 mediated α-syn fibrils dissociation was analyzed with TEM. Briefly, 10 µl of samples were diluted ten times and adsorbed on 400 mesh carbon-coated copper grids (Ted Pella, Inc, USA). Excess liquid was adsorbed by lint-free tissue paper and incubated for 1 min, following negatively stained with 2% uranyl acetate for 1 min. After the film dried, images were captured via TEM (Hitachi) with accelerating voltage at 80 kV. The length of the fibrils was measured using the open-source image processing program ImageJ.

### Dynamic light scattering (DLS) analysis

DLS experiments were performed to study the changes in the mean diameter of α-syn fibrils in the presence and absence of PFFNB2. 10 μl of fibrils were mixed with 990 μl of filtered PBS. Measurements were performed in Zetasizer Nano-ZS from Malvern Instruments with He-Ne laser. Each sample was measured in single-use polystyrene semi-micro disposable cuvettes (Fisher Emergo, Landsmeer, The Netherlands) with a path length of 1 cm. The cell holder was maintained at 25 °C for all measurements. For each sample, 10 runs were performed, with three repetitions. Data were processed using the Malvern Zetasizer Software. The error bars displayed on the DLS graphs were obtained by the SD of measurements in triplicates.

### Circular dichroism (CD) spectroscopy

The far-UV CD spectra were recorded from 195–250 nm using an Aviv model 420 spectrometer (Aviv Biomedical, Inc. Lakewood, NJ, USA). CD spectra were collected in a 1 mm path length cuvette at 25 °C with the data pitch of 1 nm. For all spectra, an average of three scans was obtained. Smoothing of CD data was done by keeping points of the window ‘5’ for removing noise from signals. The secondary structural changes in α-syn fibrils with PFFNB2 were predicted based on its negative peak at 218 nm and positive peak at 195 nm.

### Statistical analysis

All statistical analyses were performed using GraphPad Prism 8.0 and GraphPad Prism 9.3.1. Analysis of primary cortical neuron and animal experiments were performed based on at least three independent experiments. Statistical significance was determined by unpaired two-tailed Student’s *t* test or one-way ANOVA test with Tukey’s correction. *P* value lower than 0.05 was considered to indicate a significant difference.*P < 0.05, **P < 0.01, ***P < 0.001, ****P < 0.0001.

### Reporting summary

Further information on research design is available in the Nature Research Reporting Summary linked to this article.

## Supplementary information

Supplementary info

Reporting Summary

## Data Availability

PDB-4LDE and PDB-3K1K cited in this study are accessible from Protein Data Bank. All the data supporting the findings of this study are available within the paper and its supplementary information files. Source data are provided with this paper. All the DNA constructs used in this study are available upon request to the corresponding author.

## Source data

Source Data file
