# Supplementary material for: α-Synuclein fibril-specific nanobody reduces prion-like α-synuclein spreading in mice
Source: Nat Commun. 2022 Jul 19;13:4060. doi: 10.1038/s41467-022-31787-2 (PMC9296447; doi:10.1038/s41467-022-31787-2)

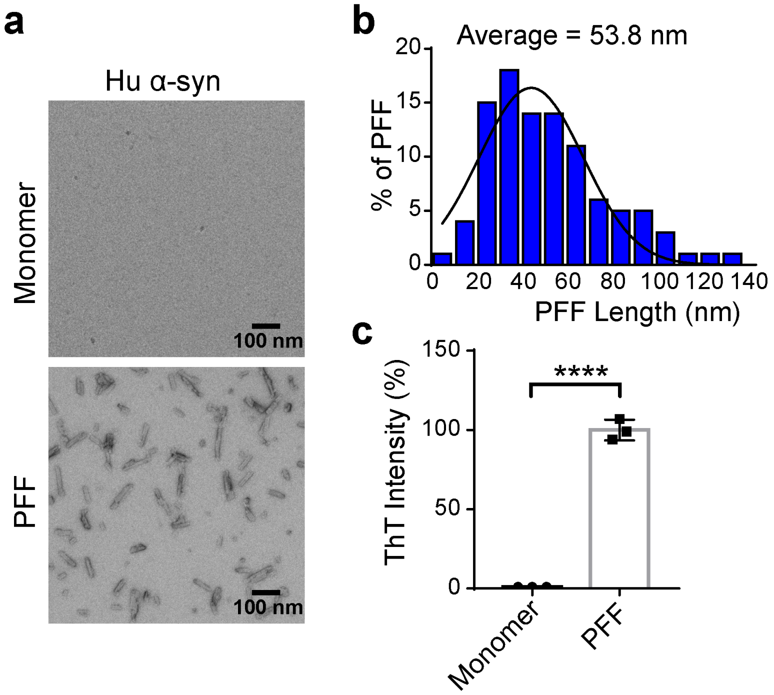

Supplementary figure 1. Characterization of α-syn monomers and PFF. a) Human α-syn monomers and PFF were characterized by transmission electron microscopy (TEM). Scale bars, 100 nm. **b)** Length distribution of human α-syn PFF. The mean length of human α-syn PFF is 53.8 nm (*n* = 237). **c)** Thioflavin T (ThT) assay for α-syn monomers and PFF. Quantification data are the means ± SEM, *n* = 3 independent experiments, *P* values were determined by two-sided Student's *t*-test (Monomer vs. PFF *P* = 0.0001). ****$P < 0.0001$. All experiments were replicated three times with similar results. Source data are provided as a Source Data file.

a

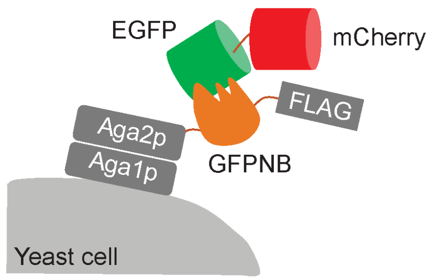

b

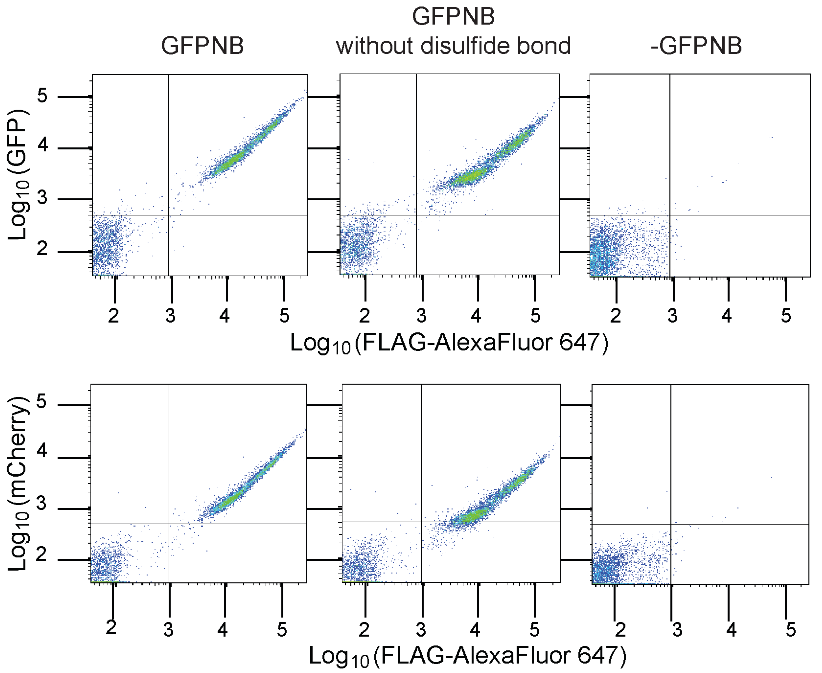

Supplementary figure 2. Testing the binding of the disulfide bond-free GFP nanobody on the yeast surface. a) Schematics of the binding assay on the yeast surface. Anti-GFP nanobody (GFPNB) with and without disulfide bond was expressed on the yeast surface following Aga2p. The yeast cells were incubated with EGFP-mCherry fusion protein. **b)** FACS analysis of yeast cells expressing GFPNB-FLAG on the yeast surface incubated with EGFP-mCherry fusion protein. GFPNB without disulfide bond (C22L, C96A mutant) retains its binding to EGFP. Negative control (-GFPNB) is yeast cells without induction therefore no GFPNB expression.

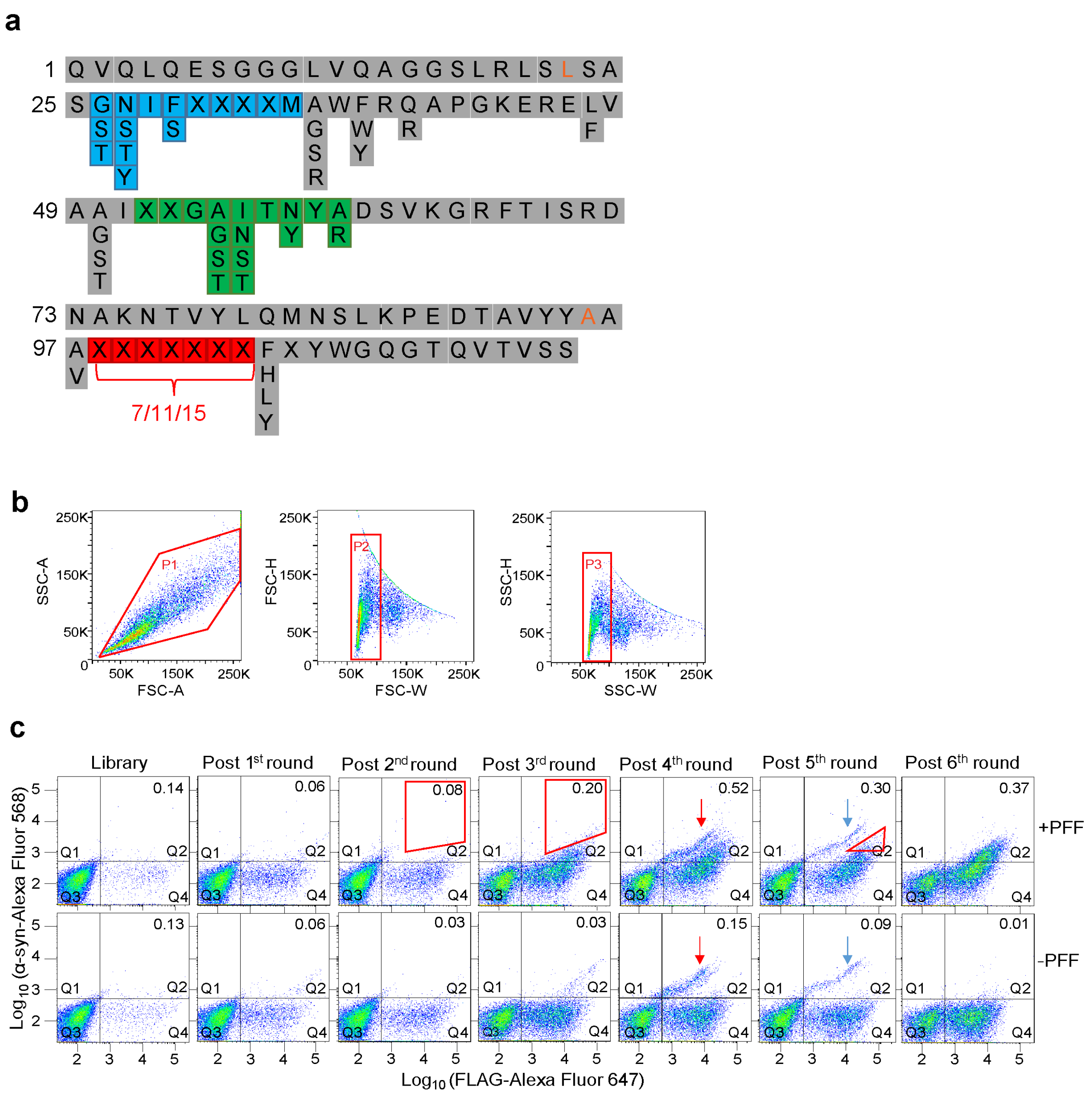

Supplementary figure 3. Amino acid sequence schematics of the nanobody library design and FACS analysis of the nanobody selection. a) Nanobody library construction based on a published protocol[34], except that the conserved cysteine residues were mutated in our library to remove the conservative disulfide bond under oxidizing conditions. C22L and C95A mutations are indicated by orange letters. The constant regions of the nanobodies were determined from the consensus sequences of the VHH from the llama gene IGHV1S1-IGHV1S1S5 and are shown in grey with only one amino acid in each position[34]. The CDR1, 2, and 3 are highlighted in blue, green, and red. X indicates site-saturated randomization with 20 amino acids. Some positions in grey have multiple amino acids in the same position, indicating randomization with those amino acids. Additionally, the CDR3 was constructed with 3 different lengths with 7, 11, or 15 amino acids randomized (red). **b)** Gating strategy to analyze single yeast cells. First, cells were plotted by FSC-A and SSC-A, and a gate P1 was drawn to include almost all the cells. Cells from P1

were then plotted by FSC-W and FSC-H and a gate between 60 - 110 FSC-W and 0 - 255 FSC-H gave population P2. Cells from P2 were then plotted by SSC-W and SSC-H and a gate between 60 - 105 SSC-W and 0 - 195 SSC-H gave population P3. Cells from population P3 were analyzed to show FLAG signal in the x-axis (640 nm laser and 670/14 emission filter) and α-syn signal in the y-axis (561 nm laser and 586/15 emission filter). **c)** FACS analysis of the rounds of nanobody selection against α-syn PFF. The red trapezoid indicates the selection gate for FACS. After one round of MACS followed by 3 more rounds of FACS, a false positive nanobody population showed up with high fluorescence signal even in the absence of α-syn PFF incubation (post 4th round). Removal of the false-positive clones on the 5th round sorting using MACS was attempted but not successful (blue arrow, post 5th round). This is possible because some low-expressing yeast cells can escape the negative selections using MACS, but will show up in the next round after re-amplification. Eventually, we used FACS to draw a tight gate to select only the true positive population, avoiding the false positive population (red triangle, post 5th round).

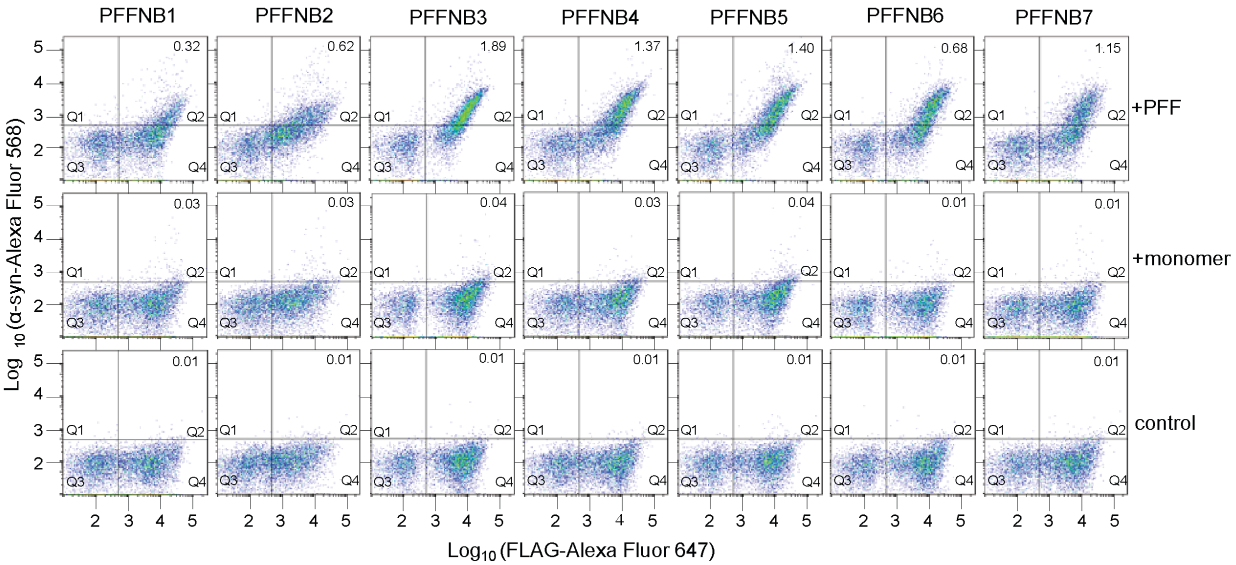
PFFNB1
PFFNB2
PFFNB3
PFFNB4
PFFNB5
PFFNB6
PFFNB7
+PFF
0.32
0.62
1.89
1.37
1.40
0.68
1.15
+monomer
0.03
0.03
0.04
0.03
0.04
0.01
0.01
control
0.01
0.01
0.01
0.01
0.01
0.01
0.01
Q1
Q2
Q3
Q4
Log 10 (α-syn-Alexa Fluor 568)
Log10 (FLAG-Alexa Fluor 647)

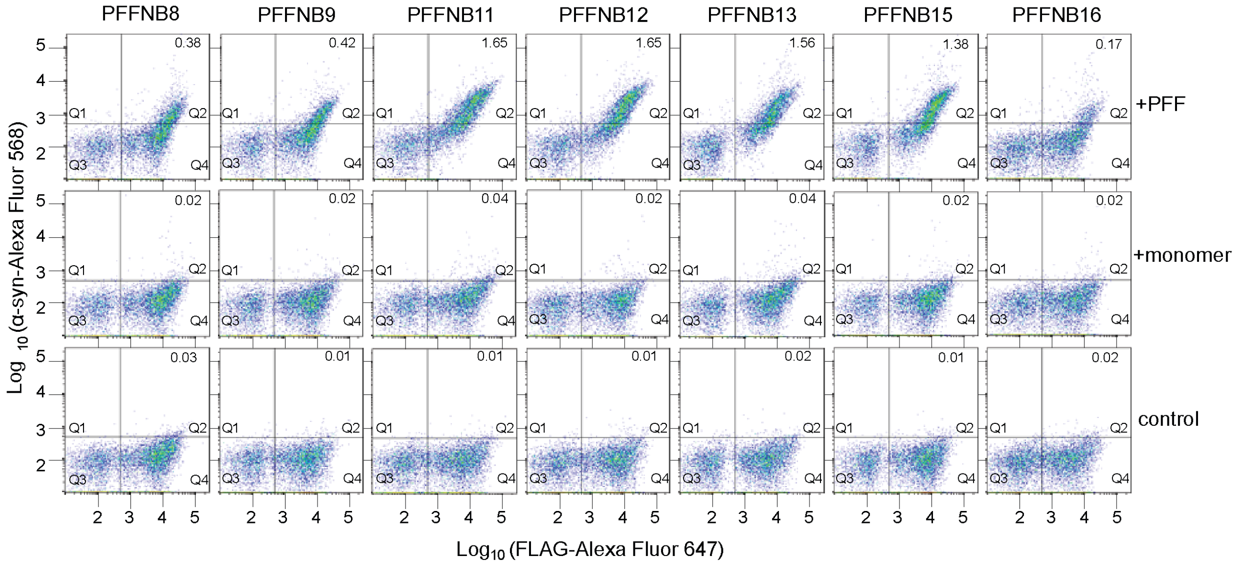
PFFNB8
PFFNB9
PFFNB11
PFFNB12
PFFNB13
PFFNB15
PFFNB16
+PFF
0.38
0.42
1.65
1.65
1.56
1.38
0.17
+monomer
0.02
0.02
0.04
0.02
0.04
0.02
0.02
control
0.03
0.01
0.01
0.01
0.02
0.01
0.02
Q1
Q2
Q3
Q4
Log 10 (α-syn-Alexa Fluor 568)
Log10 (FLAG-Alexa Fluor 647)

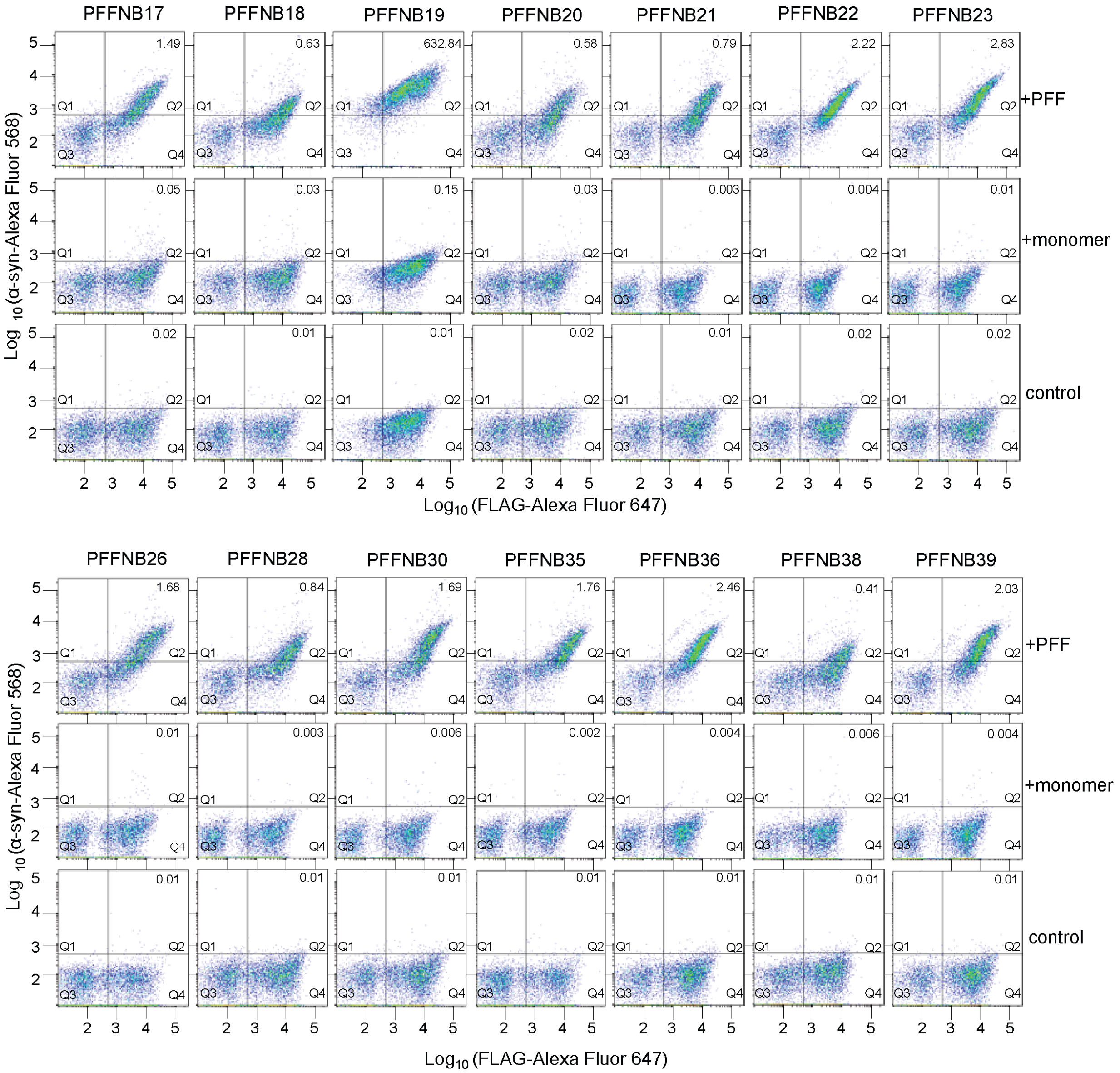

Supplementary figure 4. FACS analysis of the 28 nanobody clones selected against α-syn PFF. Yeast cells expressing 28 different nanobody clones were incubated with α-syn PFF, monomers, or just buffer (control). Then cells were then labelled with mouse anti-α-syn antibody and anti-mouse IgG antibody conjugated to AlexaFluor 568. The numbers in the upper right corner of Q2 indicate the ratio of Q2/Q4 population. All 28 clones showed selective binding to α-syn PFF over α-syn monomers.

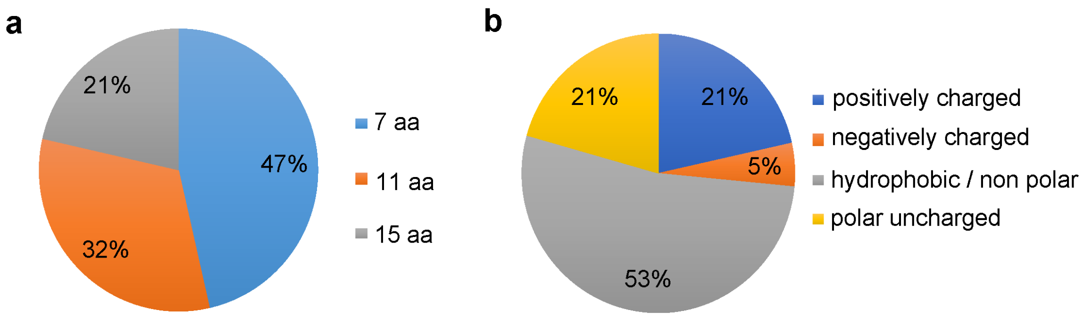

Supplementary figure 5. Analysis of the CDR3 of the 28 PFFNB clones. a) CDR3 length analysis of the 28 PFFNB clones. 47% of the nanobody clones consist of a CDR3 with 7 amino acids randomized, 32% with 11 amino acids, and 21% with 15 amino acids randomized. **b)** The CDR3 of the selected PFFNBs have a net positive charge and are rich in hydrophobic residues. Source data are provided as a Source Data file.

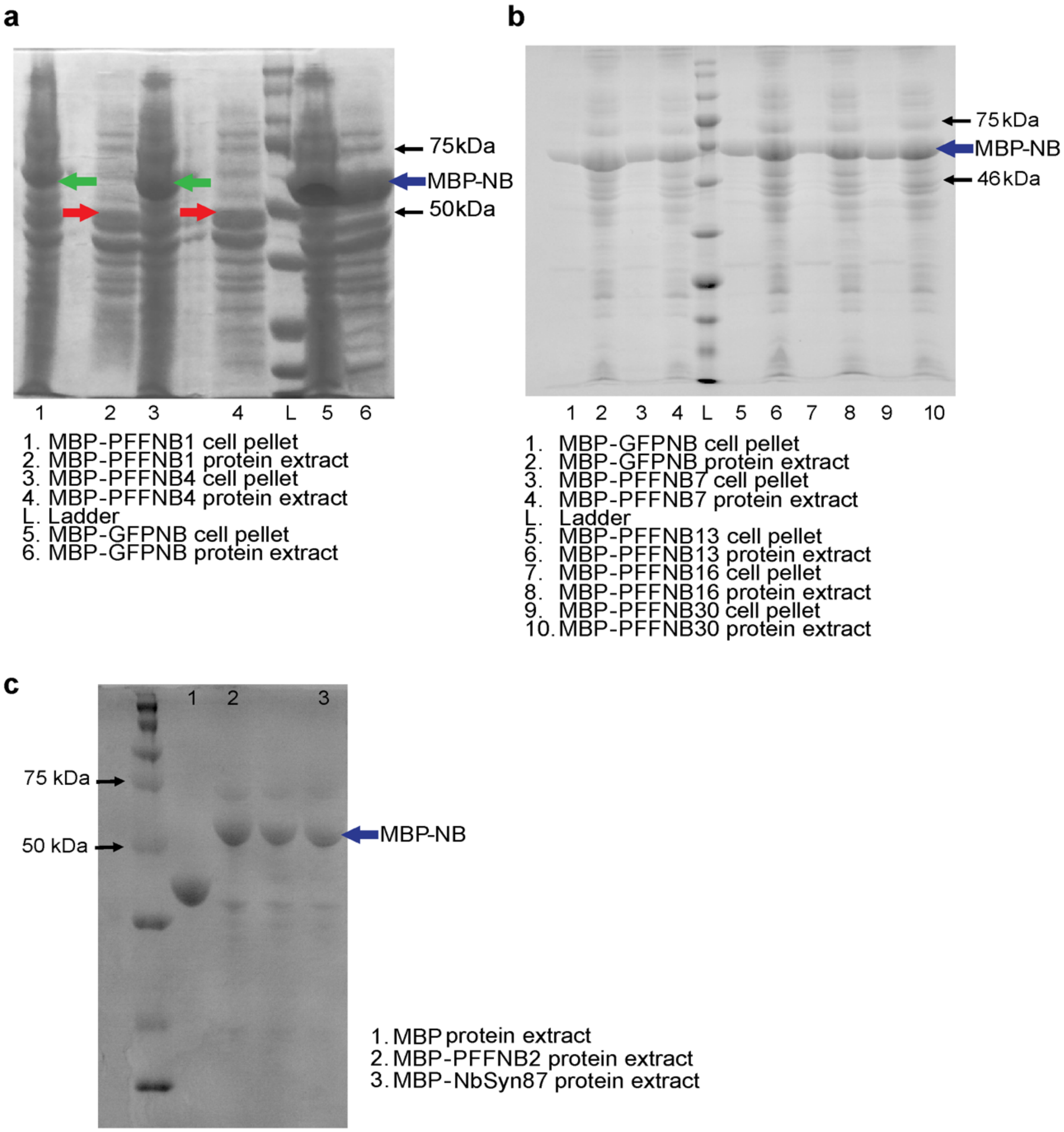

Supplementary figure 6. SDS-PAGE analysis of the PFFNBs expression and purification in *E. coli*. a) Protein expression in *E. coli* BL21. SDS-PAGE analysis of crude HisTag-MBP-PFFNBs proteins. The expected protein size is ~60 kDa. The majority of the protein at ~ 60 kDa (green arrow) was retained in the cell pellet while the extracted protein was at ~ 50 kDa (red arrow), possibly due to early termination of translation or truncation. Positive control protein HisTag-MBP-GFPNB(C22L, C96A) (positive control) appeared at the correct molecular weight (blue arrow). **b)** SDS-PAGE of HisTag-MBP-PFFNB or GFPNB expressed in *E. coli* BL21(C14) with co-expressed chaperones. All the crude PFFNB protein extract appeared at ~ 60 kDa, the correct molecular weight. A major band at ~60 kDa was also detected in the pellet. **c)** SDS-PAGE of purified HisTag-MBP, HisTag-MBP-PFFNB2, and HisTag-MBP-NbSyn87 after size exclusion chromatography, which were used in Fig. 2 and Supplementary Fig. 7. All experiments were replicated twice with similar results. Source data are provided as a Source Data file.

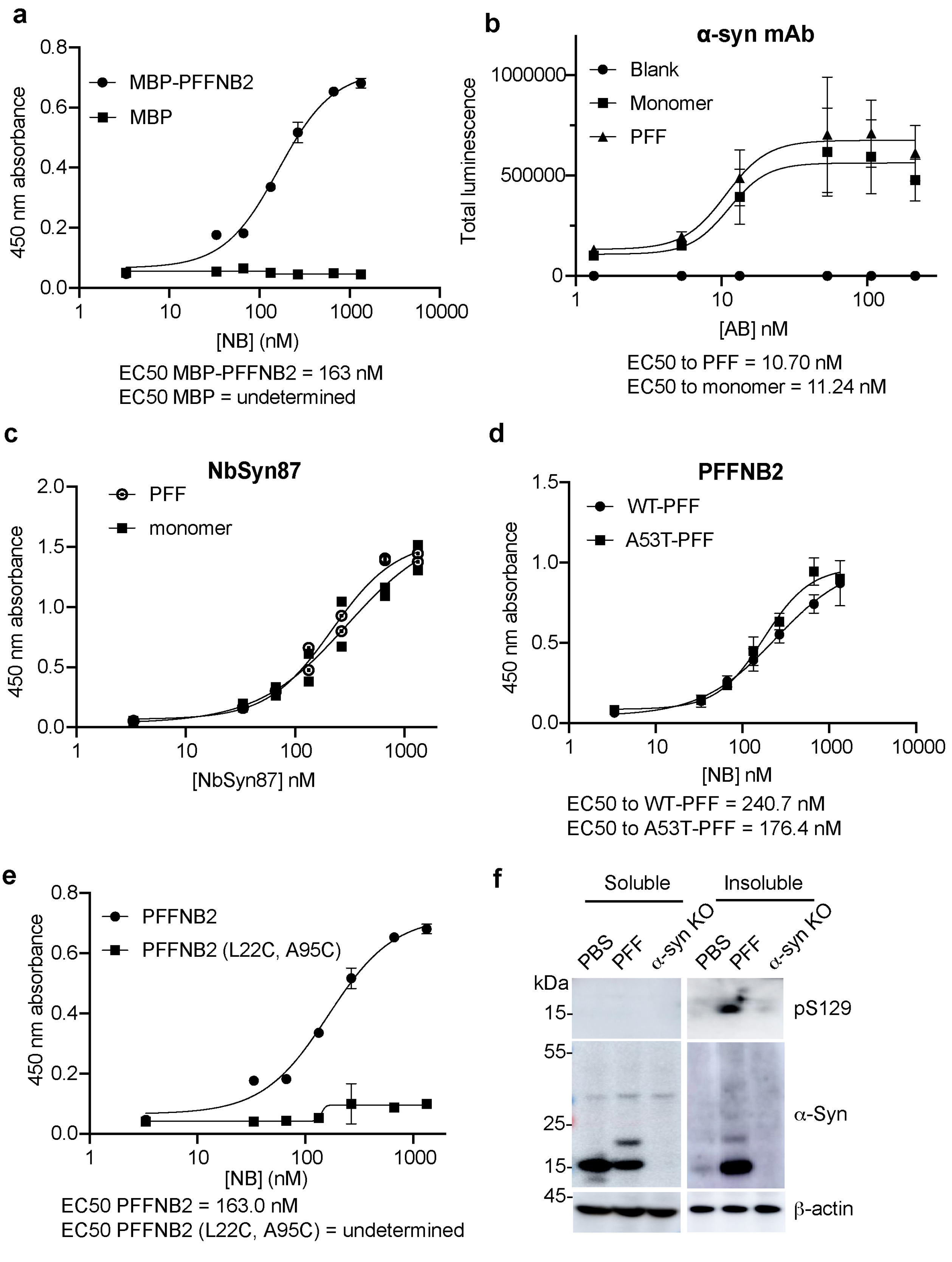
a
MBP-PFFNB2
MBP
450 nm absorbance
[NB] (nM)
EC50 MBP-PFFNB2 = 163 nM
EC50 MBP = undetermined
b
α-syn mAb
Blank
Monomer
PFF
Total luminescence
[AB] nM
EC50 to PFF = 10.70 nM
EC50 to monomer = 11.24 nM
c
NbSyn87
PFF
monomer
450 nm absorbance
[NbSyn87] nM
d
PFFNB2
WT-PFF
A53T-PFF
450 nm absorbance
[NB] nM
EC50 to WT-PFF = 240.7 nM
EC50 to A53T-PFF = 176.4 nM
e
PFFNB2
PFFNB2 (L22C, A95C)
450 nm absorbance
[NB] (nM)
EC50 PFFNB2 = 163.0 nM
EC50 PFFNB2 (L22C, A95C) = undetermined
f
Soluble
Insoluble
PBS
PFF
α-syn KO
kDa
pS129
α-Syn
β-actin

Supplementary figure 7. a) ELISA analysis of MBP-PFFNB2 and MBP binding to α-syn PFF. Wells were coated with 3 ng/µL of α-syn PFF, and then titrated with 3.3, 33.3, 66.7, 133.3, 266.7, 666.7, and 1333.3 nM of MBP-PFFNB2 or MBP alone. Three data points were collected for each concentration and shown as mean ± SEM. **b)** ELISA analysis of anti-α-syn mAb binding to α-syn monomers and PFF. Wells were coated with 3 ng/µL of α-syn PFF or monomers, and then titrated with 1.3, 5.3, 13.3, 53.5, 106.6, and 213.3 nM of anti-α-syn mAb. Three data points were collected for each concentration and shown as mean ± SEM. **c)** ELISA analysis of MBP-NbSyn87 binding to α-syn monomers and PFF. Wells were coated with 3 ng/µL of α-syn PFF or monomers, and then titrated with 3.3, 33.3, 66.7, 133.3, 266.7, 666.7, and 1333.3 nM of MBP-NbSyn87. Two data points were collected for each concentration. **d)** ELISA analysis of MBP-PFFNB2 binding to recombinant human wildtype (WT) α-syn PFF (WT-PFF) and α-syn(A53T) PFF (A53T-PFF). Wells were coated with 3 ng/µL of wild-type α-syn PFF or α-syn(A53T) PFF then titrated with 3.3, 33.3, 66.7, 133.3, 266.7, 666.7, and 1333.3 nM of MBP-PFFNB2. Three data points were collected for each concentration and shown as mean ± SEM. **e)** ELISA analysis of PFFNB2 and PFFNB2 (L22C, A95C) binding to α-syn PFF. Wells were coated with 3 ng/µL of α-syn PFF or monomers, and then titrated with 3.3, 33.3, 66.7, 133.3, 266.7, 666.7, and 1333.3 nM of MBP-PFFNB2 or MBP- PFFNB2 (L22C, A95C). Three data points were collected for each concentration and shown as mean ± SEM. **f)** Immunoblot analysis of the soluble and insoluble fractions of mice brain lysates with and without α-syn pathology, and *Snca* knock-out (KO) mice brain lysate, with indicated antibodies. All experiments above was replicated once with similar results. Source data are provided as a Source Data file.

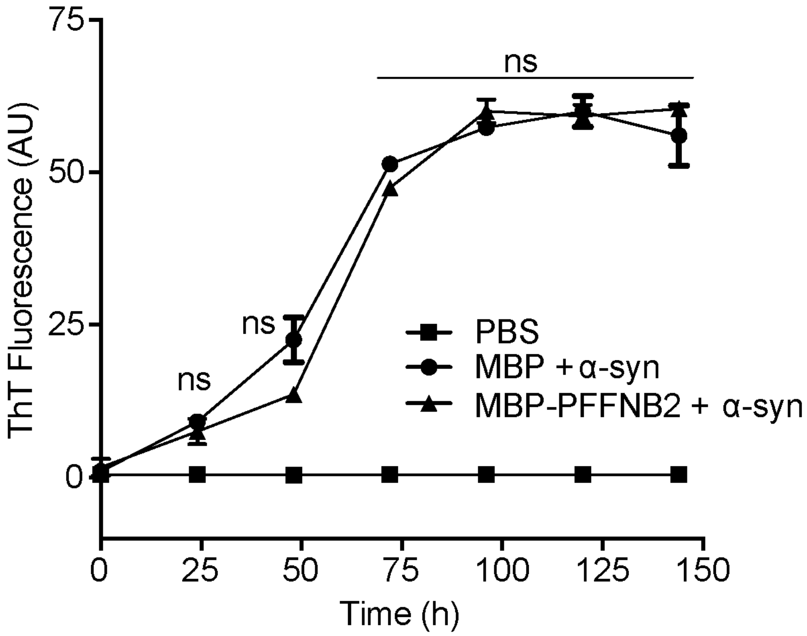

Supplementary figure 8. The ThT assay for α-syn aggregation assay with PFFNB2. Effect of MBP-PFFNB2 or MBP alone on α-syn (2 mg/mL) aggregation with the ThT assay. Quantification data are the means ± SEM, n = 3 independent experiments, P values were determined by two-sided Student's t-test. (MBP + α-syn vs. MBP-PFFNB2 + α-syn P = 0.9404). ns, not significant. Source data are provided as a Source Data file.

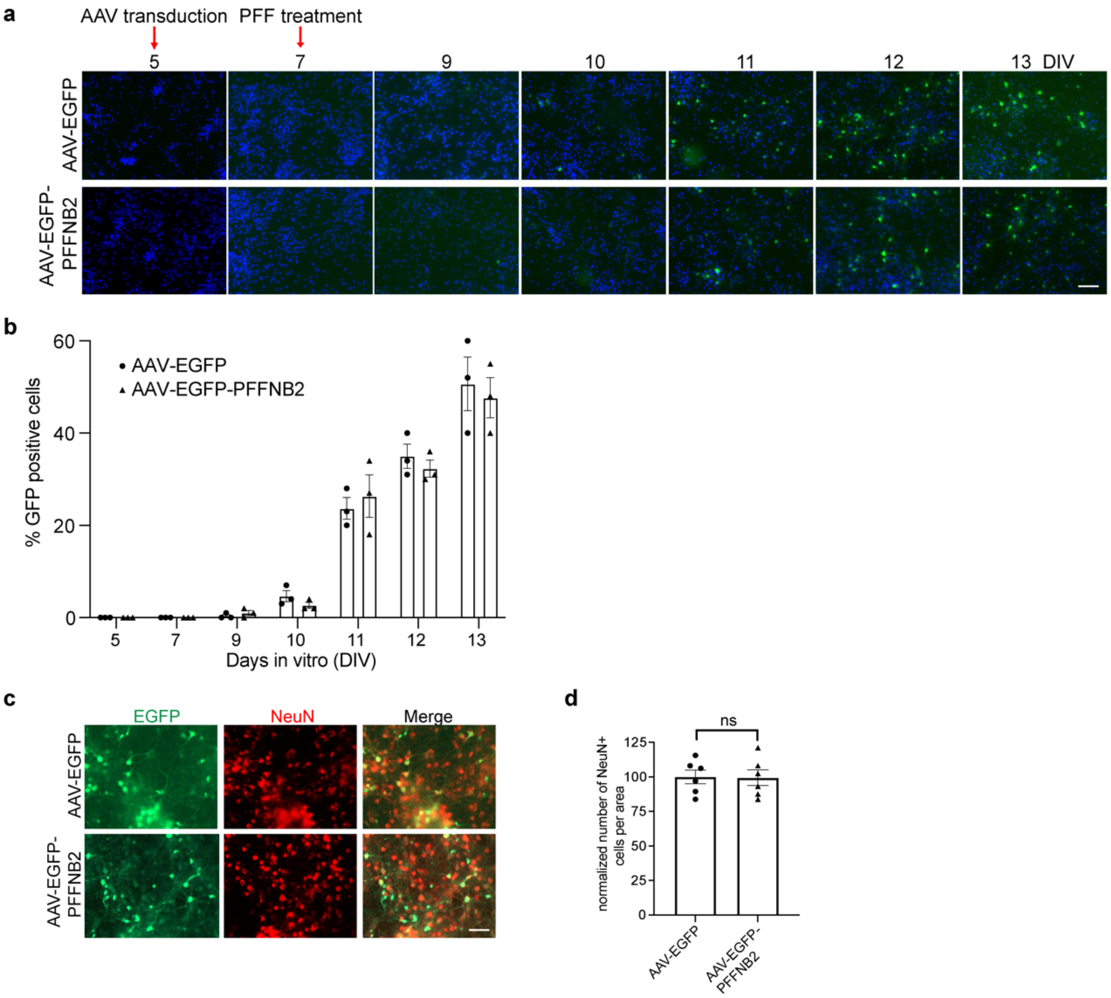

Supplementary figure 9. EGFP-PFFNB2 was expressed after PFF treatment and AAVs encoding EGFP-PFFNB2 did not cause neurotoxicity. a) WT mouse primary cortical neurons transduced with AAVs encoding EGFP (AAV-EGFP) or AAV-EGFP-PFFNB2. Cells were analyzed on indicated days *in vitro* (DIV) for EGFP expression. Red arrows indicate the day of AAV transduction and α-syn PFF administration, scale bar, 50 μm**. b)** Quantification of EGFP positive cells per 100 cells. *n* = 3 independent experiments. Quantification data are the means ± SEM. **c)** Primary cortical neurons were treated with AAVs encoding EGFP or EGFP-PFFNB2 on 5 DIV, and were assessed for neurotoxicity (NeuN immunostaining) 10 days after AAV transduction. scale bar, 50 μm. **d)** Quantification of NeuN positive cells (panel c) were counted per area and normalized to control *n* = 6 (from 3 independent experiments performed in duplicate). Quantification data are the means ± SEM, statistical significance was calculated by two-sided Student's *t*-test. (AAV-EGFP vs. AAV-EGFP-PFFNB2 *P* = 0.9392). ns, not significant. Source data are provided as a Source Data file.

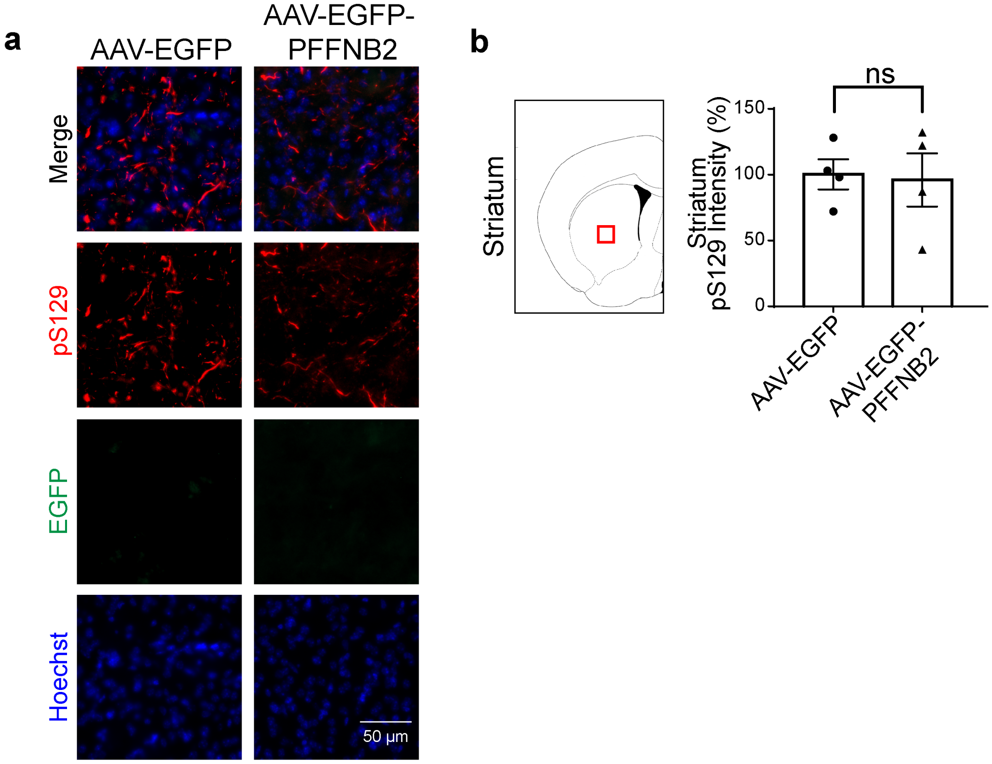

Supplementary figure 10. No significant difference in α-syn pathology in the striatum one month after striatal-PFF injection. a) The immunostaining of pS129 in the striatum of AAV-EGFP and AAV-EGFP-PFFNB2 groups. No EGFP signal can be appreciated in the striatum of both groups. Scale bar, 50 μm. **b)** Quantification of pS129 immunostaining in the striatum. Data are the means ± SEM, n = 4 mice per group, P values were determined by two-sided Student's t-test. (AAV-EGFP vs. AAV-EGFP-PFFNB2 P = 0.8605) ns, non-significant. Source data are provided as a Source Data file.

Supplementary Table 1. Amino acid sequences of identified 28 PFFNB clones

Nanobody	Sequences
PFFNB1	QVQLQESGGGLVQAGGSLRLSLSASRYIFLLQKMGWYRQAPGKERELVAGIHKGSDTNYGDSVKGRFTLSRDNAKNTVYLQMNSLKS DDTAVYYAAEPVPPRPRRRPPLPYWGQGTQVTVSS
PFFNB2	QVQLQESGGGLVQAGGSLRLSLSASRYIFTLMGMRWYRRAPGKERELVASIQVGSDTNYRDSVKGRFTLSRDNAKNTVYLQMNSLKS DDTAVYYAAAPAYARRLHRYWGQGTQVTVSS
PFFNB3	QVQLQESGGGLVQAGGSLRLSLSASRYISFLKLMGWFRRAPGKERELVAGIHNGTNTNYRDSVKGRFTLSRDNAKNTVYLQMNSLKSD DTAVYYAAEPQPLWTITVTRREHPYDYWGQGTQVTVSS
PFFNB4	QVQLQESGGGLVQAGGSLRLSPSASWYISRWWGMGWFRRAPGKERELVASIDPGGDTNYPDSVKGRFTLSRDNAKNTVYLQMNSLK SDDTAVYYAAAAHPKLPTSPGKFQYWGQGTQVTVSS
PFFNB5	QVQLQESGGGLVQAGGSLRLSLSASRYIFLLPLMGWFRRAPGKERELVAGIARGTTTYYPDSVKGRFTLSRDNAKNTVYLQMNSLKSD DTAVYYAAAHRVTKPQATKPFRYWGQGTQVTVSS
PFFNB6	QVQLQESGGGLVQAGGSLRLSLSASRNIFWWPLMGWYRRAPGKEREFVASINSGTNTYYRDSVKGRFTLSRDNAKNTVYLQMNSLKS DDTAVYYAAAPFPAPETHTYWGQGTQVTVSS
PFFNB7	QVQLQESGGGLVQAGGSLRLSLSASRYIFFWPGMGWFRRAPGKERELVASIPTGGATYYRDSVKGRFTLSRDNAKNTVYLQMNSLKS DDTAVYYAAAPPRPPATDKPLLPYWGQGTQVTVSS
PFFNB8	QVRLQESGGGLVQAGGSLRLSLSASGNIFRWWKMGWYRRAPGKERELVASIQTGANTYYADSVKGRFTLSRDNAKNTVYLQMNSLKS DDTAVYYAAEWLPSSQKNPYPHPYWGQGTQVTVSS
PFFNB9	QVQLQESGGGLVQAGGSLRLSLSASGSIFQWWAMRWFRRAPGKERELVASIHRGTVTNYPDSVKGRFTLSRDNAKNTVYLQMNSLKS DDTAVYYAAASTPDLSQNLLTDGLHRYWGQGTQVTVSS
PFFNB11	QVQLQESGGGLVQAGGSLRLSLSASRNIFLWLPMGWFRRAPGKEREFVASIGTGAATNYGDSVKGRFTLSRDNAKNTVYLQMNSLKS DDTAVYYAAARLAPHHRYSYWGQGTQVTVSS
PFFNB12	QVQLQESGGGLVQAGGSLRLSLSASRYIFPYLFMGWFRRAPGKERELVASIAAGTDTNYRDSVKGRFTLSRDNAKNTVYLQMNSLKSD DTAVYYAAARRPPKQTYLYWGQGTQVTVSS
PFFNB13	QVQLQESGGGLVQAGGCLRLSLSASRYIFHWCPMRWYRRAPGKERELVASIPRGGATYYRDSVKGRFTLSRDNAKNTVYLQMNSLKS DDTAVYYAAAHQAHPSPTATRYKYWGQGTQVTVSS
PFFNB15	QVQLQESGGGLVQAGGSLRLSLSASRYIFPWIVMGWFRRAPGKEREFVASINSGSTTNYRDSVKGRFTLSRDNAKNTVYLQMNSLKSD DTAVYYAAAQKEGRTTARTQINTYYGYWGQGTQVTVSS
PFFNB16	QVQLQESGGGLVQAGGSLRLSLSASRYIFAPRWMRWFRRAPGKERELVAGIQFGGDTNYGDSVKGRFTLSRDNAKNTVYLQMNSLKS DDTAVYYAAETLYYPRANPRPLPYWGQGTQVTVSS
PFFNB17	QVQLQESGGGLVQAGGSLRLSLSASRYIFLYPLMGWFRRAPGKEREFVASIRRGTVTNYPDSVKGRFTLSRDNAKNTVYLQMNSLKSD DTAVYYAAATGNDRCCNRLGKQMFHPYWGQGTQVTVSS
PFFNB18	QVQLQESGGGLVQAGGSLRLSLSASRYIFVWAPMGWYRRAPGKERELVASIKAGTNTYYRDSVKGRFTLSRDNAKNTVYLQMNSLKS DDTAVYYAAAAPPDETRTVLTITNDHNYWGQGTQVTVSS
PFFNB19	QVQLQESGGGLVQAGGSLRLSLSASRYIFFWPGMGWFRRAPGKERELVASIPTGGATYYRDSVKGRFTLSRDNAKNTVYLQMNSLKS

	DDTAVYYAAAPPRPPATDKPLLPYWGQGTQVTVSS
PFFNB20	QVQLQESGGGLVQAGGSLRLSLSASRYISWLQGMRWFRRAPGKEREFVASIIPGTNTNYGDSVKGRFTLSRDNAKNTVYLQMNSLKS DDTAVYYAAERRGQPLLHPYWGQGTQVTVSS
PFFNB21	QVQLQESGGGLVQAGGSLRLSLSASRYIFLPLFMGWYRRAPGKERELVAGINRGTTTNYPDSVKGRFTLSRDNAKNTVYLQMNSLKSD DTAVYYAAAAPSQTQYHKYWGQGTQVTVSS
PFFNB22	QVQLQESGGGLVQAGGSLRLSLSASRNIFSWMIMRWYRRAPGKERELVAGIKSGTDTNYRDSVKGRFTLSRDNAKNTVYLQMNSLKS DDTAVYYAAATPTQPTPHGYWGQGTQVTVSS
PFFNB23	QVQLQESGGGLVQAGGSLRLSLSASRTIFYWLGMRWFRRAPGKERELVAGIRTGATTNYRDSVKGRFTLSRDNAKNTVYLQMNSLKS DDTAVYYAAAPSVPPAYHPYWGQGTQVTVSS
PFFNB26	QVQLQESGGGLVQAGGSLRLSLSASRYIFVLPWMRWYRRAPGKERELVASITDGATTNYPDSVKGRFTLSRDNAKNTVYLQMNSLKS DDTAVYYAAEVNRPRQAYGYWGQGTQVTVSS
PFFNB28	QVQLQESGGGLVQAGGSLRLSLSASRSIFRCRYMRWFRQAPGKERELVASIICGTNTNYRDSVKGRFTLSRDNAKNTVYLQMNSLKSD DTAVYYAAETTEAHLRPVTNLMYWGQGTQVTVSS
PFFNB30	QVQLQESGGGLVQAGGSLRLSLSASRYIFWGPFMGWFRRAPGKERELVAGIAHGSNTNYADSVKGRFTLSRDNAKNTVYLQMNSLKS DDTAVYYAAAQAPVHPHHPYWGQGTQVTVSS
PFFNB35	QVQLQESGGGLVQAGGSLRLSLSASRYIFAWYRMGWFRRAPGKERELVASIQHGTITNYADSVKGRFTLSRDNAKNTVYLQMNSLKSD DTAVYYAAAQLGAPRQYRYWGQGTQVTVSS
PFFNB36	QVQLQESGGGLVQAGGSLRSLSASWNIFHFEWMRWFRRAPGKEREFVASIHRGSNTNYPDSVKGRFTLSRDNAKNTVYLQMNSLKS DDTAVYYAAAKSPFTVPLTYWGQGTQVTVSS
PFFNB38	QVQLQESGGGLVQAGGSLRLSLSASRSISWRGWMGWFRRAPGKEREFVASIGPGTNTNYADSVKGRFTLSRDNAKNTVYLQMNSLK SDDTAVYYAAEHLIPYYRFSYWGQGTQVTVSS
PFFNB39	QVQLQESGGGLVQAGGSLRLSLSASRSIFWWLGGMGWYRQAPGKERELVAGIAKGGNTNYRDSVKGRFTLSRDNAKNTVYLQMNSL KSDDTAVYYAAAQRGKSMRPLPTRRLSHSYWGQGTQVTVSS

Supplementary Table 2. Key resources table

Reagent or Resource	Source	Identifier
**Antibodies**		
Mouse anti-FLAG-Horseradish Peroxidase	Sigma-Aldrich	Cat#A8592, RRID:AB_439702
Mouse anti-α-Synuclein	BD Biosciences	Cat#610787, RRID:AB_398108
Rabbit anti-α-Synuclein	Cell Signalling	Cat#4179, RRID:AB_1904156
Rabbit anti-pS129-α-synuclein	Abcam	Cat#ab51253, RRID:AB_869973
Rabbit anti-FLAG	Sigma-Aldrich	Cat#F7425, RRID: AB_439687
Mouse anti-FLAG	Sigma-Aldrich	Cat#F3165, RRID: AB_259529
Mouse anti-NeuN	Millipore-Sigma	Cat#MAB377, RRID:AB_2298772
Goat anti-rabbit IgG-Alexa Fluor 488	Thermo Fisher Scientific	Cat#A11008, RRID:AB_143165
Goat anti-rabbit IgG-AlexaFluor 568	Thermo Fisher Scientific	Cat#A11036, RRID:AB_10563566
Goat anti-mouse IgG-AlexaFluor 568	Thermo Fisher Scientific	Cat#A11004, RRID: AB_2534072
Goat anti-mouse IgG-AlexaFluor 647	Thermo Fisher Scientific	Cat#A21235, RRID: AB_2535804
Anti-Cy5-microbeads	Miltenyi Biotech	Cat#130-042-401
Sheep anti-mouse IgG-Horseradish Peroxidase	GE Healthcare	Cat#NA931, RRID: AB_772210
Goat anti-rabbit-AlexaFluor 647	Thermo Fisher Scientific	Cat#A21245, RRID: AB_2535813
Goat anti-mouse IgG-HRP	Thermo Fisher Scientific	Cat#31430, RRID:AB_228307
**Reagent**	**Source**	**Identifier**
Hoechst 33342 Solution	Thermo Fisher Scientific	Cat#62249
DAPI	BioRad	Cat#1351303
Supersignal West Pico Plus	Thermo Fisher Scientific	Cat#34578
Pierce BCA Protein Assay	Thermo Fisher Scientific	Cat#23225
Frozen-EZ yeast Transformation Kit	Zymo Research	Cat#T2001
B-PER Bacterial Protein Extraction Reagent	Thermo Fisher Scientific	Cat#78248
Complete Mini protease inhibitor cocktail	Roche	Cat#11836170001
Halt Phosphatase inhibitor cocktail	Thermo Scientific	Cat#78420
BioPorter	Genlantis	Cat#BP502401
SuperBlock T20 (TBS)	Thermo Scientific	Cat#37536
**Mouse Strains and Cell lines**	**Source**	**Identifier**
PAC-Tg(SNCAWT)	Jackson Laboratory	Strain: 010710
C57BL/6	Charles River	Strain: 027
C57BL/6-Snca^{tm1MJMjff}/J mice	Jackson Laboratory	Strain: 016123

Software	Source	Identifier
ImageJ	NIH	https://imagej.nih.gov/ij/

Prism 8	GraphPad	https://www.graphpad.com/scientific-software/prism/
Zen lite	Zeiss	https://www.zeiss.com/microscopy/us/products/microscope-software/zen-lite.html
FlowJo	BD	https://www.flowjo.com/solutions/flowjo
Nikon NIS-Elements	Nikon	https://www.microscope.healthcare.nikon.com/products/software/nis-elements

Supplementary Table 3. Table of oligonucleotides for nanobody library construction

Oligonucleotides (Primers)	Sequence (5’ > 3’)
F1	CTTCGGTTGTCCCTGTCTGCCTCADGGWMCATCTYCNNKNNKNNKNNKATGSGCTGGTWCAGGCRGGCTCCGGGTAAAGAAAGGGAA
R1	CGTGAATCTCCCCTTCACAGAGTCTSSGTAGTWCGTADYASYACCMNNMNNTATGCYAGCGACGARTTCCCTTTCTTTACCCGGAGCC
F2	GTTCAACTCCAGGAGTCTGGTGGTGGCCTGGTTCAAGCGGGTGGGTCTCTTCGGTTGTCCCTGTCTGCCTCA
R2	CGTGAATCTCCCCTTCACAGAGTCT
F3	GAGACAACGCCAAAAATACTGTTTATCTGCAGATGAACTCTCTGAAATCCGATGATACTGCAGTATATTATGCTGCTG
R3	GACCTGGGTTCCTTGACCCCAGTAMNNGWRMNNMNNMNNMNNMNNMNNMNNTKCAGCAGCATAATATACTGCAGTATC
F4	AGACTCTGTGAAGGGGAGATTCACGTTGTCAAGAGACAACGCCAAAAATACTGTTTATC
R4	CGTCATCCTTGTAGTCGGATCCGCTGGACACTGTGACCTGGGTTCCTTGACCCCAGTA
F5	CAAGGTCTGCAGGCTAGTGGTGGAGGAGGCTCTGGTGCTAGCCAAGTTCAACTCCAGGAGTCTGGTGGTG
R5	CTCGAGCTATTACTTATCGTCGTCATCCTTGTAGTCGGATCCGCTGG
R6	GACCTGGGTTCCTTGACCCCAGTAMNNGWRMNNMNNMNNMNNMNNMNNMNNMNNMNNMNNMNNTKCAGCAGCATAATATACTGCA GTATC
R7	GACCTGGGTTCCTTGACCCCAGTAMNNGWRMNNMNNMNNMNNMNNMNNMNNMNNMNNMNNMNNMNNMNNMNNMNNTKCAGCAGCA TAATATACTGCAGTATC

Supplementary Table 4. Table of plasmid used in this study

Plasmid vector	Promoter	Host	Gene inserts	Used for	Details of gene inserts	Featured in
pCTCON2	Gal1	Yeast	Aga2p-Nanobody Library 7-FLAG	PFFNB selection	Aga2p:<br>MQLLRCFSIFSVIASVLAQELTTICEQIPSPTLESTPYSLSTTTILANG<br>KAMQGVFEYYKSVTFVSNCGSHPSTTSKGSPINTQYVF<br>Nanobody library 7:<br>QVQLQESGGGLVQAGGSLRLSLSASXXIXXXXXMXWXRXAPGKER<br>EXVAXIXXGXXTXYXDSVKGRFTLSRDNAKNTVYLQMNSLKSDDTA<br>VYYAAXXXXXXXXXXXYWGQGTQVTVSS<br>FLAG: DYKDDDDK	Nanobody selection, Fig. 1, Supplementary Fig 3, 4
pCTCON2	Gal1	Yeast	Aga2p-Nanobody Library 11-FLAG	PFFNB selection	Aga2p: idem nanobody library 7<br>Nanobody library 10:<br>QVQLQESGGGLVQAGGSLRLSLSASXXIXXXXXMXWXRXAPGKER<br>EXVAXIXXGXXTXYXDSVKGRFTLSRDNAKNTVYLQMNSLKSDDTA<br>VYYAAXXXXXXXXXXXXXXXYWGQGTQVTVSS<br>FLAG: DYKDDDDK	Nanobody selection, Fig. 1, Supplementary Fig. 3, 4
pCTCON2	Gal1	Yeast	Aga2p-Nanobody Library 15-FLAG	PFFNB selection	Aga2p: idem nanobody library 7<br>Nanobody library 14:<br>QVQLQESGGGLVQAGGSLRLSLSASXXIXXXXXMXWXRXAPGKER<br>EXVAXIXXGXXTXYXDVSVKGRFTLSRDNAKNTVYLQMNSLKSDDT<br>AVYYAAXXXXXXXXXXXXXXXXXXXYWGQGT<br>QVTVSS<br>FLAG: DYKDDDDK	Nanobody selection, Fig. 1, Supplementary Fig. 3, 4
pCTCON2	Gal1	Yeast	Aga2p-GFPNB-FLAG	Yeast surface expression	Aga2p: idem nanobody library 7<br>GFPNB:<br>QVQLVESGGALVQPGGSLRLSCAASGFPVNRYSMRWYRQAPGKE<br>REWVAGMSSAGDRSSY<br>EDSVKGRFTISRDDARNTVYLQMNSLKPEDTAVYYCNVNVGFEYW<br>GQGTQVTVSS<br>FLAG: DYKDDDDK	Supplementary Fig. 2
pCTCON2	Gal1	Yeast	Aga2p-GFPNB(C22L, C96A)-FLAG	Yeast surface expression	Aga2p: idem nanobody library 7<br>GFPNB(C22L, C96A):<br>QVQLVESGGALVQPGGSLRLSLAASGFPVNRYSMRWYRQAPGKE<br>REWVAGMSSAGDRSSYEDSVKGRFTISRDDARNTVYLQMNSLKP<br>EDTAVYYANVNVGFEYWGQGTQVTVSS<br>FLAG: DYKDDDDK	Supplementary Fig. 2
pYFJ16	T7	*E. coli*	EGFP-linker-mCherry-His	Protein purification	EGFP:<br>MVSKGEELFTGVVPILVELDGDVNGHKFSVSGEGEGDATYGKLTLK<br>FICTTGKLPVPWPTLVTTLTYGVQCFSRYPDHMKQHDFFKSAMPE<br>GYVQERTIFFKDDGNYKTRAEVKFEGDTLVNRIELKGIDFKEDGNIL<br>GHKLEYNYNSHNVYIMADKQKNGIKVNFKIRHNIEDGSVQLADHYQ<br>QNTPIGDGPVLLPDNHYLSTQSALSKDPNEKRDHMVLLEFVTAAGI<br>TLGMDELYK<br>Linker: HMGSGTGGYPYDVPDYAARDPPVAT	Supplementary Fig. 2

					mCherry:<br>MVSKGEEDNMAIIKEFMRFKVHMEGSVNGHEFEIEGEGEGRPYEG<br>TQTAKLKVTKGGPLPFAWDILSPQFMYGSKAYVKHPADIPDYLKLS<br>FPEGFKWERVMNFEDGGVVTVTQDSSLQDGEFIYKVKLRGTNFPS<br>DGPVMQKKTMGWEASSERMYPEDGALKGEIKQRLKLKDGGHYDA<br>EVKTTYKAKKPVQLPGAYNVNIKLDITSHNEDYTIVEQYE<br>RAEGRHSTGGMDELYK<br>His tag: HHHHHHH	
pYFJ16	T7	*E. coli*	His-MBP-10aa linker-TEVcs-GFPNB(C22L, C96A)-FLAG	Protein purification	His tag: HHHHHHH<br>Maltose Binding Protein:<br>MKIEEGKLVIWINGDKGYNGLAEVGKKFEKDTGIKVTVEHPDKLEEK<br>FPQVAATGDGPDIIFWAHDRFGGYAQSGLLAEITPDKAFQDKLYPF<br>TWDAVRYNGKLIAYPIAVEALSLIYNKDLLPNPPKTWEEIPALDKELK<br>AKGKSALMFNLQEPYFTWPLIAADGGYAFKYENGKYDIKDVGVDNA<br>GAKAGLTFLVDLIKNKHMNADTDYSIAEAAFNKGETAMTINGPWAW<br>SNIDTSKVNYGVTVLPTFKGQPSKPFVGVLSAGINAASPNKELAKEF<br>LENYLLTDEGLEAVNKDKPLGAVALKSYEEELAKDPRIAATMENAQ<br>KGEIMPNIPQMSAFWYAVRTAVINAASGRQTVDEALKDAQTNSSSN<br>NNNNNNNNNLGIEGRG<br>10 amino acid linker: GGSGSGSGGS<br>TEV cleavage site: ENLYFQG<br>GFPNB(C22L,C96A):<br>QVQLVESGGALVQPGGSLRLSLAASGFPVNRYSMRWYRQAPGKE<br>REWVAGMSSAGDRSSYEDSVKGRFTISRDDARNTVYLQMNSLKP<br>EDTAVYYANVNVGFEYWGQGTQVTVSS<br>FLAG: DYKDDDDK	Supplementary Fig. 6a,b
pYFJ16	T7	*E. coli*	His-MBP-10aa linker-TEVcs-PFFNB2-FLAG	Protein purification	His tag: HHHHHHH<br>Maltose Binding Protein: idem MBP-GFPNB(C22L, C96A)<br>10 amino acid linker: idem MBP-GFPNB(C22L, C96A)<br>TEV cleavage site: idem MBP-GFPNB(C22L, C96A)<br>PFFNB2:<br>QVQLQESGGGLVQAGGSLRLSLSASRYIFTLMGMRWYRRAPGKE<br>RELVASIQVGSDTNYRDSVKGRFTLSRDNAKNTVYLQMNSLKSDD<br>TAVYYAAAPAYARRLHRYWGQGTQVTVSS<br>FLAG: DYKDDDDK	Fig. 2a,b,e, Fig. 3a-e. Supplementary Fig. 6, 7a,d,e, 8
pYFJ16	T7	*E. coli*	His-MBP-10aa linker-TEVcs-NbSyn87-FLAG	Protein purification	His tag: HHHHHHH<br>Maltose Binding Protein: idem MBP-GFPNB(C22L, C96A)<br>10 amino acid linker: idem MBP-GFPNB(C22L, C96A)<br>TEV cleavage site: idem MBP-GFPNB(C22L, C96A)<br>NbSyn87:<br>QVQLQESGGGSVQTGGSLRLSCVASGYSGYMAWFRQAPGKERE<br>GIAAIYRGDKITYYAHSVQGRFTISQANAKNTVYLLMNSLKPEDTAIY<br>YCAARRVVADSPLLSKTYAYWGQGTQVTVSSFLAG: DYKDDDDK	Supplementary Fig. 7c

pYFJ16	T7	*E. coli*	His-MBP-10aa linker-TEVcs-MBP-PFFNB2(L22C,A95C)-FLAG	Protein purification	His tag: HHHHHHH<br>Maltose Binding Protein: idem MBP-GFPNB(C22L, C96A)<br>10 amino acid linker: idem MBP-GFPNB(C22L, C96A)<br>TEV cleavage site: idem MBP-GFPNB(C22L, C96A)<br>MBP-PFFNB2(L22C, A95C):<br>QVQLQESGGGLVQAGGSLRLSCSASRYIFTLMGMRWYRRAPGKERELVASIQVGSDTNYRDSVKGRFTLSRDNAKNTVYLQMNSLKSDDTAVYYCAAPAYARRLHRYWGQGTQVTVSS: DYKDDDDK	Supplementary Fig.7e
pYFJ16	T7	*E. coli*	His-MBP-FLAG	Protein purification	His tag: idem P1<br>Maltose Binding Protein: idem P1<br>FLAG: idem P1	Supplementary Fig. 3a-e, 6c, 7a, 8
pRK172	T7	*E. coli*	Human α-syn	α-syn monomer and PFF generation	Wild-type human α-syn:<br>MDVFMKGLSKAKEGVVAAAEKTKQGVAEAAGKTKEGVLYVGSKTKEGVVHGVATVAEKTK<br>EQVTNVGGAVVTGVTAVAQKTVEGAGSIAAATGFVKKDQLGKNEEGAPQEGILEDMPVDPDNEAYEMPSEEGYQDYEPEA	Throughout this study
pRK172	T7	*E. coli*	Human α-syn (A53T)	α-syn (A53T) PFF generation	Human α-syn (A53T):<br>MDVFMKGLSKAKEGVVAAAEKTKQGVAEAAGKTKEGVLYVGSKTKEGVVHGVTTVAEKTKEQVTNVGGAVVTGVTAVAQKTVEGAGSIAAATGFVKKDQLGKNEEGAPQEGILEDMPVDPDNEAYEMPSEEGYQDYEPEA	Supplementary Fig.7d
pLX208 (without hygromycin resistance gene)	CMV	Mammalian	EGFP-linker-PFFNB2	HEK293T cell infection	EGFP:<br>MVSKGEELFTGVVPILVELDGDVNGHKFSVSGEGEGDATYGKLTLKFICTTGKLPVPWPTLVTTLTYGVQCFSRYPDHMKQHDFFKSAMPEGYVQERTIFFKDDGNYKTRAEVKFEGDTLVNRIELKGIDFKEDGNILGHKLEYNYNSHNVYIMADKQKNGIKVNFKIRHNIEDGSVQLADHYQQNTPIGDGPVLLPDNHYLSTQSALSKDPNEKRDHMVLLEFVTAAGITLGMDELYK<br>Linker: GSGATNGSGSGGGAP<br>PFFNB2: idem MBP-PFFNB2	Fig. 2c-d
pLX208 (with hygromycin resistance gene)	CMV	Mammalian	Human α-syn (A53T)	HEK293T cell infection	Human α-synuclein(A53T):<br>MDVFMKGLSKAKEGVVAAAEKTKQGVAEAAGKTKEGVLYVGSKTKEGVVHGVTTVAEKTKEQVTNVGGAVVTGVTAVAQKTVEGAGSIAAATGFVKKDQLGKNEEGAPQEGILEDMPVDPDNEAYEMPSEEGYQDYEPEA	Fig. 2c-d
AAV2	synapsin	Mammalian	EGFP-linker-PFFNB2	Expression in neuron culture and mouse brain	EGFP:<br>MVSKGEELFTGVVPILVELDGDVNGHKFSVSGEGEGDATYGKLTLKFICTTGKLPVPWPTLVTTLTYGVQCFSRYPDHMKQHDFFKSAMPEGYVQERTIFFKDDGNYKTRAEVKFEGDTLVNRIELKGIDFKEDGNILGHKLEYNNSHNVYIMADKQKNGIKVNFKIRHNIEDGSVQLADHYQQNTPIGDGPVLLPDNHYLSTQSALSKDPNEKRDHMVLLEFVTAAGITLGMDELYK<br>Linker: GSGATNGSGSGGGAP<br>PFFNB2: idem MBP-PFFNB2	Fig. 3f-g, Fig. 4, Fig. 5, Supplementary Fig. 9, 10

AAV2	synap sin	Mammal ian	EGFP	Expression in neuron culture and mouse brain	EGFP: idem EGFP-PFFNB2	Fig. 3f-g, 4, 5, Supplementa ry Fig. 9, 10

Supplementary method

Here's an illustration to construct the synthetic nanobody DNA Library 7 that consists of a CD3 with 7 amino acids randomized. First, CDR1 and 2 were constructed using overlapping PCR with degenerated primers forward primer 1 (F1) and reverse primer 1 (R1). The overlapped PCR segment were then amplified using primers F2 and R2. CDR 3 DNA fragment was constructed using overlapping PCR with primer F3 and degenerated primer R3. The overlapped PCR segment was amplified using primers F4 and R4. Finally, to assemble the whole nanobody construct, CDR 1, 2 DNA segment and CDR3 DNA segment were joined together using overlapping PCR. The overlapped DNA fragment of the complete nanobody gene was further amplified using primers F5 and R5. For the other two libraries with 11 and 15 amino acids randomized in CDR3, degenerated primer R3 was replaced with R6 and R7, respectively.

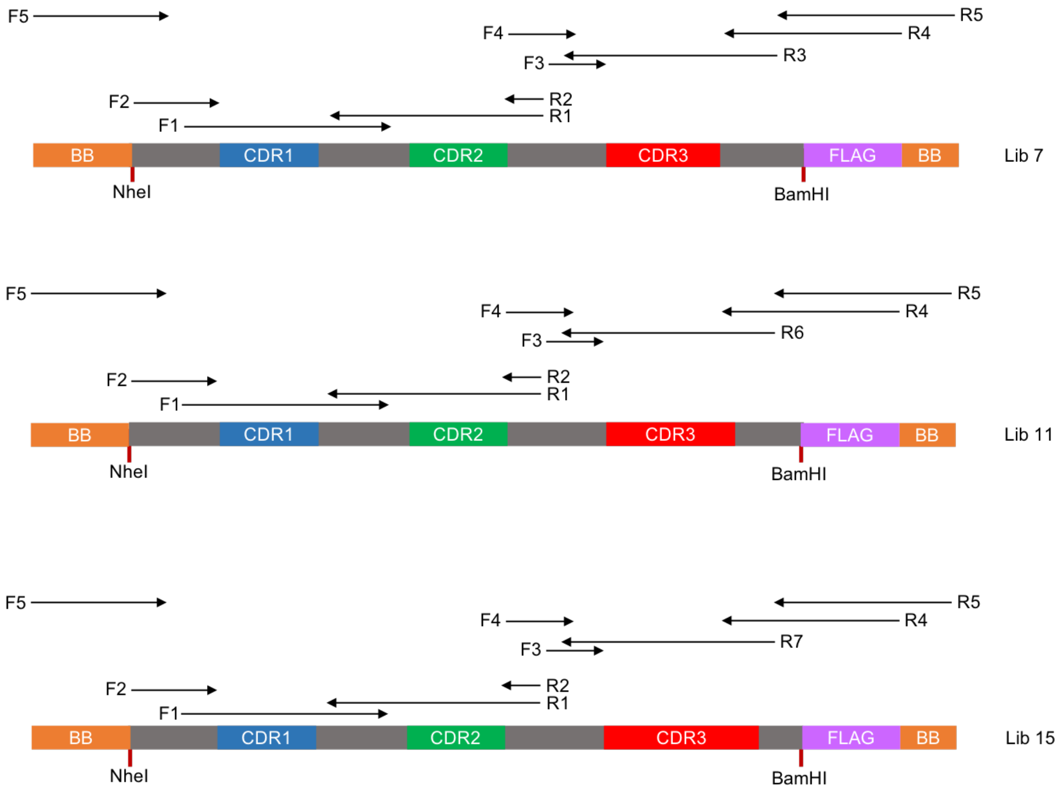

Supplement: Supplementary file 1 — Supplementary info [file 41467_2022_31787_MOESM1_ESM.pdf]
