# Supplementary material for: α-Synuclein fibril-specific nanobody reduces prion-like α-synuclein spreading in mice
Source: Nat Commun. 2022 Jul 19;13:4060. doi: 10.1038/s41467-022-31787-2 (PMC9296447; doi:10.1038/s41467-022-31787-2)
Supplement: Supplementary file 2 — Reporting Summary [file 41467_2022_31787_MOESM2_ESM.pdf]

nature portfolio

Corresponding author(s): Wenjing Wang, PhD. and Xiaobo Mao, PhD.

Last updated by author(s): Jun 1, 2022

Reporting Summary

Nature Portfolio wishes to improve the reproducibility of the work that we publish. This form provides structure for consistency and transparency in reporting. For further information on Nature Portfolio policies, see our Editorial Policies and the Editorial Policy Checklist.

Statistics

For all statistical analyses, confirm that the following items are present in the figure legend, table legend, main text, or Methods section.

n/a	Confirmed	
☐	☒	The exact sample size (*n*) for each experimental group/condition, given as a discrete number and unit of measurement
☐	☒	A statement on whether measurements were taken from distinct samples or whether the same sample was measured repeatedly
☐	☒	The statistical test(s) used AND whether they are one- or two-sided<br>*Only common tests should be described solely by name; describe more complex techniques in the Methods section.*
☐	☒	A description of all covariates tested
☐	☒	A description of any assumptions or corrections, such as tests of normality and adjustment for multiple comparisons
☐	☒	A full description of the statistical parameters including central tendency (e.g. means) or other basic estimates (e.g. regression coefficient) AND variation (e.g. standard deviation) or associated estimates of uncertainty (e.g. confidence intervals)
☐	☒	For null hypothesis testing, the test statistic (e.g. F, t, r) with confidence intervals, effect sizes, degrees of freedom and P value noted<br>*Give P values as exact values whenever suitable.*
☒	☐	For Bayesian analysis, information on the choice of priors and Markov chain Monte Carlo settings
☒	☐	For hierarchical and complex designs, identification of the appropriate level for tests and full reporting of outcomes
☐	☒	Estimates of effect sizes (e.g. Cohen's d, Pearson's r), indicating how they were calculated

Our web collection on statistics for biologists contains articles on many of the points above.

Software and code

Policy information about availability of computer code

Data collection	BD FACSDiva, Nikon NIS-Elements v5.11.03, imageQuant LAS 4000mini scanner, Zeiss Axio Observer Z1, Gen5 v3.11, Malvern Zetasizer Software
Data analysis	ImageJ 1.53a, GraphPad Prism 8.0, GraphPad Prism 9.3.1(350), FlowJo 10, Nikon NIS-Elements v5.11.03, Zen lite 2012 (Blue edition), Malvern Zetasizer Software, Amersham Image 6000

For manuscripts utilizing custom algorithms or software that are central to the research but not yet described in published literature, software must be made available to editors and reviewers. We strongly encourage code deposition in a community repository (e.g. GitHub). See the Nature Portfolio guidelines for submitting code & software for further information.

Data

Policy information about availability of data

All manuscripts must include a data availability statement. This statement should provide the following information, where applicable:

- Accession codes, unique identifiers, or web links for publicly available datasets
- A description of any restrictions on data availability
- For clinical datasets or third party data, please ensure that the statement adheres to our policy

All the data supporting the findings of this study are available within the paper and its supplementary information files. All the DNA constructs used in this study are available upon request to the corresponding author.

Field-specific reporting

Please select the one below that is the best fit for your research. If you are not sure, read the appropriate sections before making your selection.

☒ Life sciences ☐ Behavioural & social sciences ☐ Ecological, evolutionary & environmental sciences

For a reference copy of the document with all sections, see nature.com/documents/nr-reporting-summary-flat.pdf

Life sciences study design

All studies must disclose on these points even when the disclosure is negative.

Sample size	10,000 to 20,000 yeast cells were analyzed for the FACS experiments for determining nanobody's binding to alpha-synuclein preformed fibrils.<br>For the ELISA experiments, two data points were collected for each concentration. Each set of experiment was repeated 2-3 times.<br>For the experiments in HEK293T cells, 10-20 fields of views were analyzed for each condition, each of which contains more than 30 cells. >100 alpha-synuclein puncta were analyzed for Pearson's correlation analysis.<br>For the experiments in the primary cortical neurons, 15-25 fields of view were conducted for each condition, each of which contains more than 200 neurons.<br>For in vitro fibril dissociation assay, all measurements were performed in triplicate.<br>For yeast, ELISA and HEK293T and dissociation assay experiments, samples are determined to be adequate based on literatures describing similar experiments to give fair comparison to control and reproducibility.<br>For the experiments in vivo, 4 mice were analyzed for each group. Sample size for animal experiment was determined to be adequate based on literature describing similar experiments and consistency of measurable difference between the groups.
Data exclusions	No data was excluded
Replication	All applicable experiments are replicated with similar results. Details of each experiment are described in the figure legends.
Randomization	Cells and animals were randomly assigned to different experimental groups.
Blinding	The investigators were not blinded. The data was collected and analyzed objectively with no bias.

Reporting for specific materials, systems and methods

We require information from authors about some types of materials, experimental systems and methods used in many studies. Here, indicate whether each material, system or method listed is relevant to your study. If you are not sure if a list item applies to your research, read the appropriate section before selecting a response.

Materials & experimental systems

n/a	Involved in the study
☐	☒ Antibodies
☐	☒ Eukaryotic cell lines
☒	☐ Palaeontology and archaeology
☐	☒ Animals and other organisms
☒	☐ Human research participants
☒	☐ Clinical data
☒	☐ Dual use research of concern

Methods

n/a	Involved in the study
☒	☐ ChIP-seq
☐	☒ Flow cytometry
☒	☐ MRI-based neuroimaging

Antibodies

Antibodies used	We provided the list of all antibodies including the supplier, catalog number and Research Resource Identifiers (RRID) number in Table S2 (Supplementary data)
Validation	Antibodies used in this study are validated by manufacturer, RRIDs and in the cited articles.

Eukaryotic cell lines

Policy information about cell lines

Cell line source(s)	HEK 293T/17 cell line (ATCC, cat#: CRL-11268)
Authentication	HEK 293T/17 cell line was authenticated based on morphology, cell replicate rate and cell transfection efficiency every 3 months. No misidentified cell line was used in this study.

Mycoplasma contamination	The cell line tested negative against mycoplasma contamination using DAPI DNA staining.
Commonly misidentified lines (See ICLAC register)	No misidentified cell line was used in this study.

Animals and other organisms

Policy information about studies involving animals; ARRIVE guidelines recommended for reporting animal research

Laboratory animals	PAC-Tg(SNCAWT) mice (Strain: 010710) and C57BL/6-Sncatm1MJMjff/J mice (strain: 016123) were obtained from the Jackson Laboratory (3 male, 1 female per group). Mice were injected with α-syn PFF at the age of two months and sacrificed at the age of three months. Pregnant CD-1 mice (Strain: 022) were used for primary cortical neuron cultures (embryonic age 15.5 days) (Charles River). All mice were housed under standard condition of constant temperature of (22±1 degree celcius), relative humidity of 42%, and 12 hour light cycle with food and water.
Wild animals	No wild animals were used.
Field-collected samples	No field-collected samples were used.
Ethics oversight	All procedures were carried out with approval from the University Committee on Use and Care of Animals at the Johns Hopkins University

Note that full information on the approval of the study protocol must also be provided in the manuscript.

Flow Cytometry

Plots

Confirm that:

- [x] The axis labels state the marker and fluorochrome used (e.g. CD4-FITC).
- [x] The axis scales are clearly visible. Include numbers along axes only for bottom left plot of group (a 'group' is an analysis of identical markers).
- [x] All plots are contour plots with outliers or pseudocolor plots.
- [x] A numerical value for number of cells or percentage (with statistics) is provided.

Methodology

Sample preparation	EBY100 yeast cells, immunostained with corresponding antibodies described in method section
Instrument	BD FACS Aria III Cell Sorter
Software	BD FACSDiva and FlowJo
Cell population abundance	Gated single-cell yeast population for analysis in P3 (described below) represents >90% of the total population.
Gating strategy	FACSAria III Cell Sorter equipped with 640 nm laser and 670/14 emission filter (for Alexa Fluor647) as well as 561 nm laser and 586/15 emission filter (for Alexa Fluor568) were used for yeast cell analysis and sorting. To analyze single yeast cells, cells were plotted by FSC-A and SSC-A, and a gate P1 was draw to include almost all the cells. Cells from P1 were then plotted by FSC-W and FSC-H and a gate between 60 - 110 FSC-W and 0 - 255 FSC-H gave population P2. Cells from P2 were then plotted by SSC-W and SSC-H and a gate between 60 - 105 SSC-W and 0 - 195 SSC-H gave population P3. Cells from population P3 were analyzed to show FLAG signal in the x-axis (640 nm laser and 670/14 emission filter) and α-syn signal in the y-axis (561 nm laser and 586/15 emission filter).

- [x] Tick this box to confirm that a figure exemplifying the gating strategy is provided in the Supplementary Information.
